# Antidiabetic Potential of Novel 1,3,5-Trisubstituted-2-Thioxoimidazloidin-4-One Analogues: Insights into α-Glucosidase, α-Amylase, and Antioxidant Activities

**DOI:** 10.3390/ph15121576

**Published:** 2022-12-17

**Authors:** Salma M. Khirallah, Heba M. M. Ramadan, Hossam Aladl Aladl Aladl, Najla O. Ayaz, Lina A. F. Kurdi, Mariusz Jaremko, Samar Zuhair Alshawwa, Essa M. Saied

**Affiliations:** 1 Biochemistry Department, Faculty of Science, Port Said University, Port Said 42526, Egypt; 2 Chemistry Department, Faculty of Science, Port Said University, Port Said 42526, Egypt; 3 Department of Internal Medicine, Faculty of Medicine, AL-Azhar University, Nasr City, Cairo 11651, Egypt; 4 Department of Biochemistry, College of Science, University of Jeddah, Jeddah 80327, Saudi Arabia; 5 Department of Biology, College of Science, University of Jeddah, Jeddah 80327, Saudi Arabia; 6 Smart-Health Initiative and Red Sea Research Center, Division of Biological and Environmental Sciences and Engineering, King Abdullah University of Science and Technology, Thuwal 23955, Saudi Arabia; 7 Department of Pharmaceutical Sciences, College of Pharmacy, Princess Nourah bint Abdulrahman University, P.O. Box 84428, Riyadh 11671, Saudi Arabia; 8 Chemistry Department, Faculty of Science, Suez Canal University, Ismailia 41522, Egypt; 9 Institute for Chemistry, Humboldt Universität zu Berlin, Brook-Taylor-Str. 2, 12489 Berlin, Germany

**Keywords:** substituted-2-thiohydantoins, diabetes mellitus, antidiabetic activity, α-glucosidase activity, α-amylase activity, cytotoxicity, antioxidant activity, molecular docking simulation

## Abstract

As the ninth leading cause of death globally, diabetes mellitus (DM) is considered to be the worst chronic metabolic disease requiring an enormous need for healthcare with over 578 million expected cases by 2023. Several recent findings have demonstrated that mediating the activity of carbohydrate-hydrolyzing enzymes, including α-amylase and α-glucosidase, could be a potential strategy for managing the development of DM. In the presented study, a novel set of 1,3,5-trisubstituted-2-thioxoimidazolidin-4-ones was designed, synthesized, and characterized. The antidiabetic activity of the synthesized compounds was explored by assessing their inhibitory activity toward α-amylase and α-glucosidase enzymes. The results demonstrated that this class of compounds exhibits considerable inhibitory activity toward both α-amylase and α-glucosidase enzymes. Among the synthesized compounds, compound **5a** demonstrated the most inhibitory activity with IC_50_ of 5.08 and µg/mL and 0.21 µg/mL toward α-glucosidase and α-amylase activities, respectively, as compared to the drug Acarbose (IC_50_ = 5.76 µg/mL and 0.39 µg/mL, respectively). To gain insights into the antidiabetic potential of compound **5a**, we assessed the cytotoxic and antioxidant activities. Our findings indicated that compound **5a** displays considerable cytotoxicity toward WI-38 cells with an IC_50_ of 88.54 µg/mL, as compared to the drug Celecoxib (IC_50_ = 93.05 µg/mL). Further, compound **5a** exhibited a high scavenging activity toward 2,2-Diphenyl1-picrylhydrazyl (DPPH) free radicals (IC_50_ = 51.75 µg/mL) and showed a low potential to produce ROS as indicated by the monitoring of the generated H_2_O_2_ (132.4 pg/mL), as compared to Trolox (IC_50_ = 58.09 µg/mL) and Celecoxib (171.6 pg/mL). Finally, we performed extensive molecular modeling studies to affirm the binding affinity of this class of compounds to the binding pocket of α-amylase and α-glucosidase enzymes. Collectively, our findings indicate that this class of compounds, particularly compound **5a**, could be utilized as a lead structure for the development of novel compounds with potential antidiabetic and antioxidant activities.

## 1. Introduction

With more than 578 million expected cases globally by 2023, diabetes mellitus (DM) is considered to be the worst chronic metabolic disease requiring enormous healthcare costs and a high mortality rate (ninth leading cause of global death) [1]. The pancreas secretes insulin which reduces glucose synthesis and enhances glucose absorption from the circulatory system into peripheral tissues such as skeletal muscle and adipose tissue. In diabetic patients, developed insulin resistance (T2DM) and/or insufficient secreted insulin (T1DM) cause hyperglycemia which is characterized by elevated glucose levels in the blood [2]. Hyperglycemia is considered to be an independent risk factor for the development of macrovascular complications, which basically damage organs (eye, kidney, nervous system) and cause life-threatening implications [3]. Further, hyperglycemia has been implicated in the development of gangrene, stroke, and cardiovascular diseases. Several recent findings have demonstrated that mediating the activity of carbohydrate-hydrolyzing enzymes, including α-amylase and α-glucosidase, could be a potential strategy for managing the development of DM [4]. These enzymes are mainly responsible for the cleavage of carbohydrates to small sugar units leading to elevated glucose blood levels. Pancreatic α-amylase hydrolyzes larger carbohydrate units by cleaving α-D-(1,4)-glycosidic linkages into oligosaccharides, which subsequently are subjected to further hydrolysis by the α-glucosidase enzyme to afford small glucose units in the blood [5]. Upregulation of α-amylase and α-glucosidase activities has been correlated to postprandial hyperglycemia and the development of DM [6]. Regulation of the α-glucosidase and α-amylase activities manipulates postprandial hyperglycemia associated with a high-carbohydrates diet by avoiding the rapid elevation in plasma glucose levels [7,8]. Accordingly, targeting α-glucosidase and α –amylase enzymes with small-molecule antagonists has received a great deal of attention [9]. To date, the only clinically available carbohydrate-hydrolyzing enzyme inhibitors to mediate postprandial hyperglycemia are limited to the carbasugars, including the common enzyme inhibitors of miglitol, voglibose, and acarbose [10]. These drugs act as competitive inhibitors to natural carbohydrates and efficiently bind to the active pocket of the enzymes with high-binding affinities [11]. While miglitol and voglibose exhibit selectivity to block the α-glucosidase enzyme, acarbose demonstrates a dual-inhibitory activity toward the α-amylase and α-glucosidase enzymes [12]. Although these drugs have succeeded in effectively reducing postprandial hyperglycemia, they present several adverse effects including gastrointestinal side effects such as diarrhea, nausea, and bloating, necessitating the discovery of a novel class of antagonists with dual-inhibitory activity that are safe for long-term usage [13,14]. Moreover, iminosugars are simple and small structure molecules purely linked to carbohydrates, with significant pharmacological potential. These glycomimetics’ broad range of bioactivities, combined with their amazing drug profile, make them desirable therapeutic applicants for a variety of medical treatments. The ability of iminosugars to act as antagonists of carbohydrate-processing enzymes suggests that they could be used to treat hyperglycemia, as they possess effective α- and β-glucosidase inhibition [15,16]. 

Several studies have demonstrated that the generation of reactive oxygen species (ROS) contributes to the biological activity and the mode of action of bioactive and pharmacological compounds, [17]. ROS-producing compounds (such as doxorubicin and menadione) possess pharmaceutical benefits as potent anticancer and anti-inflammatory drugs [18,19]. On the other hand, ROS production is related to the pathogenesis of several diseases including cardiovascular, neurodegenerative, cancer, and diabetic diseases [20]. Therefore, the bioactive compounds which induce the production of ROS have been considered as inappropriate leads in the drug discovery [21]. The ROS family, including free radicals (such as OH^•^ and O^•−2^) and non-radicals (such as H_2_O_2_), are mainly reactive species produced by the metabolism of oxygen. Hyperglycemia induces several specific signaling and metabolic pathways which play a crucial role in the production of ROS, oxidative stress, and cell death [22]. ROS, including hydrogen peroxide, hydroxyl radical, and superoxide anion, mediate several cellular processes by regulating the activity of enzymes involved in signal transduction [23]. Accumulation of ROS is related to the pathogenesis of several diseases including cardiovascular, neurodegenerative, cancer, and diabetic diseases [24]. Increased ROS production causes oxidative stress, which causes a number of cellular alterations. Acute and chronic elevated levels of glucose in diabetes increase ROS generation and invoke apoptosis in β-cells [25]. Therefore, the discovery of a novel class of antidiabetic compounds with potential free radical scavenging properties is considered to be an appealing approach for DM therapy.

**Figure 1:**
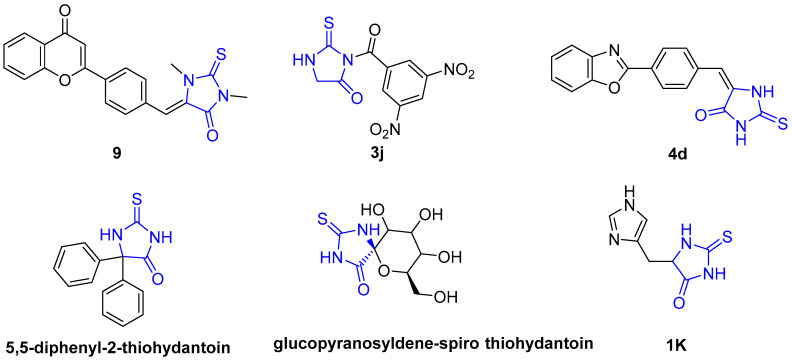
Representative structures of 2-thioxoimidazolidin-4-one analogues with potential antioxidant and antidiabetic activities.

Heterocycles are a class of compounds which possess one or more hetero atoms (O, N, or S) that have been extensively explored in diverse pharmaceutical applications. As a crucial component in several bioactive natural and medicinal compounds, these heterocycles have attracted considerable attention from synthetic researchers in the last decades [26,27,28]. Among heterocycles, 2-thioxoimidazolidin-4-ones (2-thiohydantoin) have received significant consideration owing to their potential and broad-spectrum biological and medicinal activities. This class of five-membered heterocycles is characterized by the presence of a reactive cyclic thiourea core and possesses several positions for substitution (N1, N3, and C5) [29]. A simple substitution on the 2-thiohydantoin scaffold can result in a variety of biological effects, such as anticancer, anti-inflammatory, antimicrobial, antidiabetic, antiviral, and antioxidant activity [30,31,32]. To date, the antidiabetic potential of 2-thiohydantoin scaffold has not been fully explored; however, several studies have demonstrated the antidiabetic activity of 2-thiohydantoin. Early in 1970 it was first reported that 5,5-diphenyl-2-thiohydantoin displays the ability to significantly inhibit the thyroxine-induced activation of liver enzymes, including the malic enzyme, glucose-6-phostphatase, and pyruvate carboxylase (Figure 1) [33]. The exact mode of inhibitory action has not been fully understood; however, it was suggested that 5,5-diphenyl-2-thiohydantoin acts similarly on these enzymes. Glucopyranosylidene-spiro-thiohydantoin demonstrated potential anti-T2D activity by targeting the activity of glycogen phosphorylase leading to diminishing glucose blood levels and sensitizing cells toward streptozotocin [34,35]. Further, several generations of flavonyl-substituted-2-thiohydantoin analogues have been synthesized which demonstrated the ability to increase the level of insulin release in cells [36,37]. Among reported compounds, Compound **9** exhibited a substantial capacity to increase the release of insulin in INS-1 cells (40% increase, as compared to control) at 10 µg/mL concentration in the presence of 5.6 mmol/L glucose (Figure 1) [36]. Recently, 2-thiohydantoins have been recognized as potential inhibitors toward the activity of carbohydrate-hydrolyzing enzymes, including α-amylase and α-glucosidase. The original unsubstituted-2-thiohydantoin (C_3_H_3_N_2_SO) displayed a moderate inhibitory activity toward α-amylase and α-glucosidase activities with IC_50_ of 410.35 µg/mL and 356.22 µg/mL, respectively [38]. Interestingly, the substitution of a 2-thiohydantoin scaffold was seen to be beneficial in improving the inhibitory activity of this class of heterocycles. Qamar et al. reported the inhibitory activity of a novel set of N3-(substituted benzoyl)-2-thiohydantoins toward α-amylase and α-glucosidase activities and demonstrated that substitution at the N3-position significantly influenced the inhibitory activity of this class of compounds. Among the reported analogues, Compound **3j** exhibited the most potent inhibitory activity toward α-glucosidase and α-amylase enzymes (IC_50_ = 51 and 8.2 µM, respectively) (Figure 1) [39]. Further, Singh et al. reported the synthesis and inhibitory activity of benzoxazolyl-based benzylidene-C5-substituted-2-thiohydantoins as rhodanine analogues. The authors showed that, among the synthesized analogues, Compound **4** considerably inhibited the activity of the α-amyloglucosidase enzyme (IC_50_ = 18.43 µM) (Figure 1) [40]. It should also be noted that the antidiabetic activity of the 2-thiohydantoin scaffold has been supported by the findings as possessing substantial antioxidant activity. Several studies have reported the potential activity of 2-thiohydantoin analogues to significantly scavenge free radicals, suggesting the potential antioxidant property [41,42]. 

**Figure 2:**
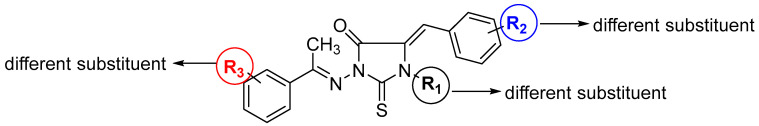
The design and examined structural features of novel 1,3,5-trisubstituted-2-thiohydantoin derivatives.

As previously discussed, the antidiabetic and antioxidant potential of the 2-thiohydantion scaffold has mainly been focused on N3 and/or C5-substituted scaffolds. However, the detailed structural features and antidiabetic activity of the trisubstituted-2-thiohydantoin scaffold are still unexplored. Continuing our efforts to synthesize bioactive compounds [43,44,45,46,47,48,49,50,51], in the present study we report the design and synthesis of novel class of 1,3,5-trisubstiuted-2-thiohydantoins. Although mono-and disubstituted 2-thiohydantion scaffolds have previously been reported as α-glucosidase and α-amylase inhibitors, the structural features around the trisubstituted-scaffold have not yet been explored. Accordingly, in our study we aimed to investigate and optimize the structural features of the 1,3,5-trisubstituted scaffold (Figure 2). The antidiabetic potency of synthesized compounds was explored by evaluating their inhibitory activity toward α-amylase and α-glucosidase. Further, the antioxidant activity of synthesized compounds was assessed by evaluating their potential to scavenge free radicals and to produce reactive oxygen species. Finally, we performed detailed in silico molecular docking simulation studies to examine the binding mode of this class of compounds toward the active pockets of α-amylase and α-glucosidase enzymes. 

## 2. Results and Discussion

**Scheme 1:**
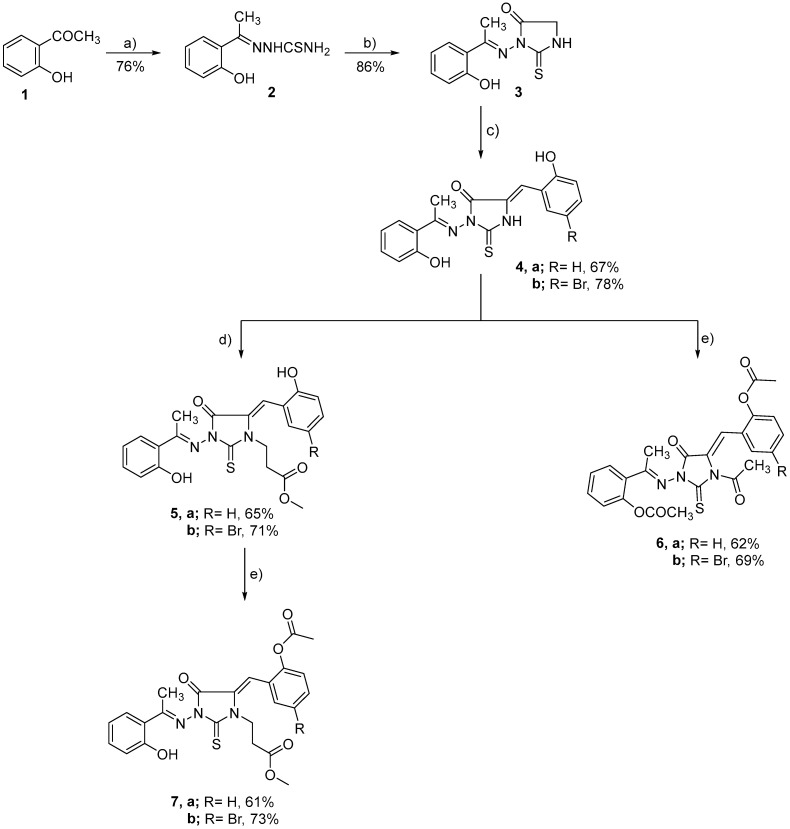
Synthesis of 1,3,5-trisubstituted-2-thioxohydantoin analogues (**3**–**7**). Reaction conditions and reagents: (**a**) Thiosemicarbazide, AcOH, ethanol, reflux, 12 h; (**b**) Ethyl chloroacetate, AcONa, ethanol, reflux, 16 h; (**c**) 2-Hydroxybenzaldehyde or 5-bromo-2-hydroxybenzaldehyde, AcONa, dimethylformamide, reflux, 12–16 h; (**d**) Methyl acrylate, triethylamine, dimethylformamide, reflux, 12–14 h; (**e**) Acetic anhydride, reflux, 12–16 h.

Several facile and efficient synthetic approaches have been developed for the synthesis of 2-thiohydantoins which allowed different structural variations at different positions [43,52,53,54]. In the current study, we designed and synthesized a number of novel 2-thiohydantoin analogues following our previously reported synthetic approach [43,44,51]. The detailed synthesis of 1,3,5-trisubstituted-2-thioxoimidazolidin-4-one derivatives (**3–7**) is illustrated in Scheme 1. The synthetic approach started from the commercially available 2-hydroxy acetophenone (**1**). Condensation of 2-hydroxy acetophenone with thiosemicarbazide in the presence of catalytic acetic acid afforded the corresponding thiosemicarbazone analogue (**2**) in a satisfactory yield (76%). The formation of the 2-thioxoimidazolidine-4-one scaffold was successfully achieved by the reaction of thiosemicarbazone **2** with ethyl chloroacetate in the presence of fused AcONa to afford 3-(2-hydroxyphenyl ethylidene) amino-2-thioxoimidazolidin-4-one (**3**) in a good yield. To explore the structural feature of the 2-thioxoimidazolidin-4-one scaffold at position C5, we reacted compound **3** with different aromatic aldehydes (mainly; 2-hydroxybenzaldehye and 4-bromo-2-hydroxybenzaldehyde) in the presence of AcONa to furnish 5-arylidene-3-(2-hydroxy-phenylethylidene)-amino-2-thioxoimidazolidine-4-ones (**4a**–**b**) in 67–78% yields. With compounds **4a**–**b** in hand, we extended our investigations to explore the structural features at position N1. Thus, compounds **4a**–**b** were subjected to acetic anhydride under reflux to afford 5-arylidene-3-(2-acetoxyphenyl ethylidene)-amino-2-thioxo-1-acetyl imidazolidine-4-ones (**6a**–**b**) in good yields (62–69%). Further, compounds **4a**–**b** were reacted with methyl acrylate in the presence of triethylamine to give [5-arylidene-4-oxo-3-(2-hydroxyphenylethylidene) amino-2-thioxoimidazolidin-1-yl] methyl propionate **5a**–**b** (65–71% yields). Finally, the effect of phenolic hydroxyl groups was examined by reacting compounds **5a**–**b** with acetic anhydride under standard conditions to afford the corresponding mono-acetate methyl [5-arylidene-4-oxo-3-phenylethylidene)-amino-2-thioxoimidazolidin-1-yl]-propionate (**7a**–**b**) analogues in 61–73% yields. 

The structure of all synthesized compounds was explored and elucidated by a set of analytical analysis including FT-IR, ^1^H-NMR, elemental analysis, and ^13^C-NMR analysis. The 2-thiohydantoin (**3**) showed four singlet signals at δ 12.59, 12.14, 4.02, and 2.52 ppm in the ^1^HNMR spectrum which were assigned to the protons of hydroxyl (OH), NH, methylene proton of imidazole ring, and methyl groups, respectively. The aromatic protons appeared in their respective region as multiplet signals (131.91, 129.59, 119.56, 119.37, and 117.50 ppm). The ^13^C-NMR spectrum revealed four signals at δ 174.05, 168.44, 153.57, and 159.52 ppm which referred to the thiocarbonyl (C=S), carbonyl (C=O), azomethine (C=N), and (C-O) groups, respectively. While the carbon signals of methylene of the imidazole ring and a methyl group were observed at δ 33.99 and 14.81 ppm. The structure of compounds **4a**–**b** was characterized by the appearance of new proton signals at δ 10.51 and 10.84 ppm which assigned the new hydroxyl groups, and the disappearance of the signal at δ 4.02 ppm of the methylene protons (NCH_2_CO) in the imidazole ring of the compound **3**. The proton signals of hydroxy (OH) and NH groups in compounds **4a**–**b** appeared as singlet signals at δ 12.42, 12.70, and 12.31, 12.74 ppm, respectively. Additionally, the ^1^HNMR spectra of **4a**–**b** displayed the H-olefinic (-CH=) as a singlet signal at δ 7.96. The structure of compounds **5a**–**b** was demonstrated by the disappearance of the proton signals of the NH group at δ 12.70 and 12.74 ppm, and appearance of three proton signals at δ 4.18, 2.83, and 3.62 ppm (two triplet signals and singlet signal) which were assigned to the NCH_2_CH_2_COOCH_3_ moiety. Further, the ^13^C-NMR spectra of compounds **5a**–**b** displayed four carbon signals at δ 168.68–168.92, 5.14–52.66, 39.11, 39.34, and 31.26–31.68 ppm, which referred to the carbon signals of carbonyl of ester, methoxy (OCH_3_), and two methylene (CH_2_) groups. The ^1^H -NMR spectra of compounds **6a**–**b** were demonstrated by the absence of the proton signals at δ 12.31–12.75 and 10.51–10.85 ppm for the two hydroxyl (OH) and NH groups, and the appearance of proton signals at δ 1.90–2.70 ppm which were assigned to the acetyl (NCOCH_3_) and two acetoxy (OCOCH_3_) groups. In addition, the ^13^C-NMR spectra of compounds **6a**–**b** revealed carbon signals of N-acetyl (NCOCH_3_) and two acetoxy (OCOCH_3_) at δ 162.12–168.20 and 20.00–22.15 ppm. The ^1^H-NMR spectra of compounds **7a**–**b** demonstrated the disappearance of the proton signals for hydroxy groups at δ 10.57 and 10.92 ppm, and the appearance of two singlet signals at δ 2.02–2.45 ppm which were assigned to the methyl protons (CH_3_) of the acetoxy groups (OCOCH_3_). 

### 2.1. Assessment of α-Glucosidase Inhibitory Activity 

**Figure 3:**
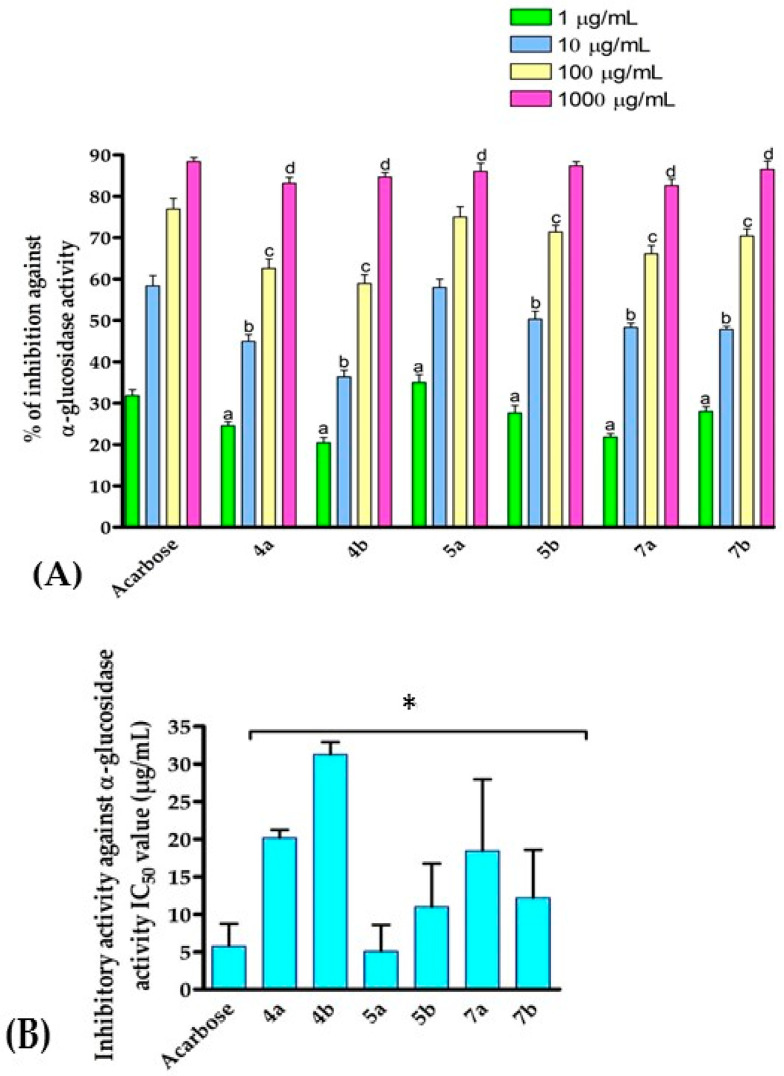
Inhibitory activities of compounds **4a**–**b**, **5a**–**b**, and **7a**–**b** against α-glucosidase activity (n = 4). (**A**) Percentage of inhibition. (**B**) IC_50_ values (μg/mL). Data are presented as mean ± SD. a *p* < 0.05 versus acarbose at concentration 1 μg/mL, b *p* < 0.05 versus acarbose at concentration 10 μg/mL, c *p* < 0.05 versus acarbose at concentration 100 μg/mL, d *p* < 0.05 versus acarbose at concentration 1000 μg/mL,* *p* < 0.05 versus acarbose for the IC_50_ value.

The *α*-glucosidase enzyme is mainly expressed in the small intestine and catalyzes the breakdown of carbohydrates into glucose units by cleaving the glucosidic bonds [55,56]. *α*-Glucosidase blockers are the first-line drugs for the treatment of non-insulin-dependent DM by suppressing carbohydrate hydrolysis and consequently retarding glucose absorption into the bloodstream and reducing postprandial hyperglycemia [57,58]. *α*-Glucosidase is, thus, considered to be an attractive pharmaceutical target for the treatment of hypoglycemia or lactic acidosis via control of the blood glucose levels [59,60]. Accordingly, there is a continuous need to develop a potential class of *α*-glucosidase inhibitors. Toward this aim, we explored the potency of the synthesized compounds to inhibit the activity of *α*-glucosidase. We examined the inhibitory activity of the synthesized compounds following the reported assay by Peytam et al. [10]. Thus, the compounds at different concentrations (1000, 100, 10, and 1 µg/mL) were incubated with *α*-glucosidase and subsequently *p*-nitrophenyl-β-D-glucopyranoside was added and the absorbance was monitored at 405 nm. In our evaluations, Acarbose, a potent antidiabetic drug, has been utilized as a pharmacological reference drug [61]. As depicted in Figure 3, the results indicated that compounds **4a**–**b**, **5a**–**b**, and **7a**–**b** exhibited a considerable inhibitory activity toward *α*-glucosidase activity at different concentrations. The N3, C5-disubstituted 1,3-thiohydantoins (**4a** and **4b**) exhibited a moderate inhibitory activity toward *α*-glucosidase enzyme. Thus, the introduction of 2-vinylphenol moiety to the C5-position of the 1,3-thiohydantoin scaffold provided a compound (**4a**) with IC_50_ of 20.17 µg/mL. Bromination of compound **4a** at position 4 in the 2-vinylphenol moiety noticeably diminished the inhibitory activity of the compound (**4b**, IC_50_ ~31.28 µg/mL), indicating that the introduction of bromine significantly impaired the binding of the compound into the active site of the *α*-glucosidase enzyme. To further investigate the structural aspects of 1,3-thiohydantoin scaffold, a substitution at the N1-position with the methyl-proponate group was performed. Interestingly, the introduction of the methyl-proponate group at the N1-position substantially improved the inhibitory activity of the compound (**5a** and **5b**). Again, the 4-bromo-2-vinylphenol analogue (**5b**) exhibited a lower inhibitory activity as compared to the 2-vinylphenol analogue (**5b**) with IC_50_ of 11.02 µg/mL and 5.76 µg/mL, respectively. Finally, acylation of compound **5a** provided a compound (**7a**) with considerably impaired inhibitory activity (IC_50_ = 18.41 µg/mL), suggesting the role of the phenolic-OH groups in the inhibitory activity of compound **5a**. On the other hand, acylation of compound **5b** did not provide a profound effect on the inhibitory activity of the compound (**7b**, IC_50_ = 12.22 µg/mL). These findings are in accordance with previous reports which revealed that 1,3-thiohydantoin analogues exhibit a potent inhibitory activity toward *α*-glucosidase enzyme [38,40,42]. Acarbose is a complex oligosaccharide which has been FDA-approved as an antidiabetic drug for the treatment of T2DM by targeting *α*-glucosidase and *α*-amylase enzymes [58,62]. Nevertheless, it has adverse gastrointestinal effects including diarrhea and flatulence. Thus, the discovery of a novel class of potent antidiabetic agents is urgently demanded. Among the examined compounds, compound **5a** exhibited the most inhibitory activity toward *α*-glucosidase activity (IC_50_ = 5.76 µg/mL), compared to that of Acarbose (IC_50_ = 5.76 µg/mL). Our results indicate that 1,3,5-trisubstituted-thiohydantoins could be considered as a promising class of *α*-glucosidase inhibitors with the potential to be antidiabetic agents.

### 2.2. Assessment of α-Amylase Inhibitory Activity 

**Figure 4:**
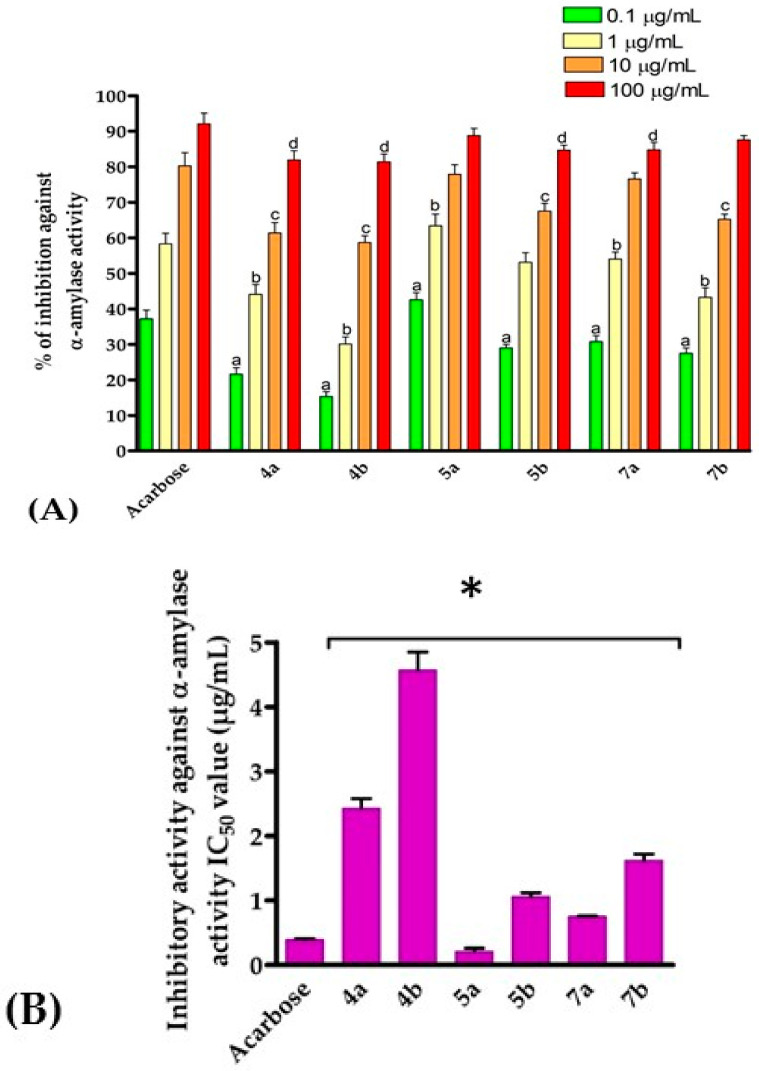
Inhibitory activity of compounds **4a**–**b**, **5a**–**b**, and **7a**–**b** against α-amylase activity (n = 4). (**A**) Percentage of inhibition. (**B**) IC_50_ values (μg/mL). Data are presented as mean ± SD. a *p* < 0.05 versus acarbose at concentration 0.1 μg/mL, b *p* < 0.05 versus acarbose at concentration 1 μg/mL, c *p* < 0.05 versus acarbose at concentration 10 μg/mL, d *p* < 0.05 versus acarbose at concentration 100 μg/mL,* *p* < 0.05 versus acarbose for the IC_50_ value.

Next, we further explored the antidiabetic activity of the synthesized compounds by examining their inhibitory activity toward α-amylase activity. α-Amylase is secreted in the salivary glands and pancreas and typically catalyzes the hydrolysis of the α-1,4-glycosidic linkage of starch to smaller oligosaccharides [63,64]. Furthermore, pancreatic α-amylase mediates the activity of glycoproteins in the small intestine membrane, such as sucrase-isomaltase and sodium/glucose cotransporter 1, via binding with *N*-linked oligosaccharides [65]. Upregulation of α-amylase activity has been demonstrated to raise blood sugar levels leading to diabetes [66]. Several α-amylase antagonist-based drugs have been clinically applied to regulate the glucose blood level and the associated T2DM progression [67]. However, attempts to find novel antagonists with enhanced effectiveness and minimum adverse effects are still worth investigating. In this regard, we examined the inhibitory activity of the synthesized compounds **4a**–**b**, **5a**–**b**, and **7a**–**b** toward α-amylase activity. Therefore, compounds at different concentrations (100, 10, 1, and 0.1 µg/mL) were mixed with the α-amylase enzyme and subsequently 3,5-dinitrosalicylic acid was added and the absorption was assessed at 595 nm. The inhibitory activities of the synthesized compounds and Acarbose (reference drug) are presented in Figure 4. The results showed that the synthesized compounds possessed a substantial inhibitory activity toward α-amylase activity. Interestingly, the inhibitory activity of the compounds toward α-amylase activity was in harmony with that of the *α*-glucosidase activity. The structural elements accountable for the inhibitory activity of the compounds toward *α*-glucosidase activity were in compliance with the inhibitory activity of the compounds toward α-amylase activity. For example, the introduction of 2-vinylphenol moiety to the 2-thiohydantoin scaffold at the C5 position provided a compound with a considerable inhibitory activity (compound **4a**, IC_50_ = 2.43 µg/mL). Substitution at the N1 position significantly improved the potency of the compound as observed for compounds **5a** (IC_50_ = 0.21 µg/mL). Further, the introduction of bromine to the 2-vinylphenol moiety demonstrated an impairment effect on the activity of the compound, as shown in compounds **4b** (IC_50_ = 4.57 µg/mL), and **5b** (IC_50_ = 1.06 µg/mL). Acylation of phenolic-OH diminished the activity of the compound as indicated for compounds **7a** (IC_50_ = 0.75 µg/mL) and **7b** (IC_50_ = 1.62 µg/mL), suggesting the role of the phenolic-OH in the binding of the 2-thiohydantoin scaffold to the active pocket of α-amylase enzyme. The dual inhibition of α-amylase and *α*-glucosidase activities has been considered an effective strategy for the development of therapeutical drugs for DM. To date, several potent natural and synthetic antagonists with dual inhibitory activities toward α-amylase and *α*-glucosidase enzymes have been reported which succeeded in being applied in clinics [63,64,68]. These drugs, however, suffer from several side effects, necessitating the development of novel and effective antagonists. Among the synthesized and evaluated compounds, compound **5a** and **7a** showed the best dual-inhibitory activities toward both α-amylase (IC_50_ = 0.21 µg/mL and 0.75 µg/mL, respectively) and *α*-glucosidase enzymes (IC_50_ = 5.08 µg/mL and 18.41 µg/mL, respectively) compared to that of the FDA-approved antidiabetic drug Acarbose. These findings again indicated that the trisubstituted-2-thiohydantoin scaffold could be considered for the development of potential antidiabetic compounds with a dual-antagonist effect toward *α*-glucosidase and α-amylase activities.

### 2.3. Assessment of In Vitro Cytotoxicity Activity against WI-38 Cells

**Figure 5:**
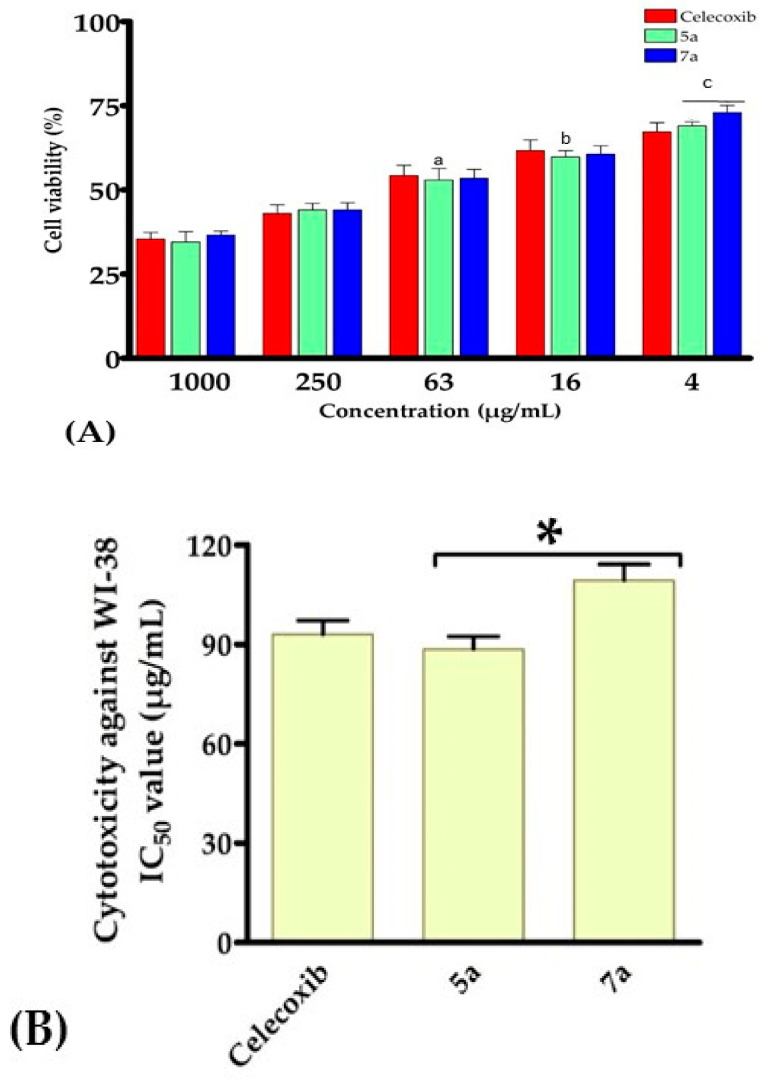
In vitro cytotoxicity of compounds **5a** and **7a** against WI-38 cell line (n = 4). (**A**) Percentage of cell viability in different concentrations. (**B**) IC_50_ values (μg/mL). Data are presented as mean ± SD. a *p* < 0.05 versus celecoxib at concentration (63 μg/mL), b *p* < 0.05 versus celecoxib at concentration (16 μg/mL), c *p* < 0.05 versus celecoxib at concentration (4 μg/mL) * *p* < 0.05 versus celecoxib for the IC_50_ value.

Based on the promising dual inhibitory activity of this class of compounds, we next evaluated the cytotoxicity of compounds **5a** and **7a** against human lung fibroblast cell line WI-38 in order to gain insights into the toxicity of this class of compounds utilizing the MTT assay [43,44,45,69]. In this regard, lung fibroblast cells were cultured and treated with compounds **5a** and **7a** for 48 h at different concentrations (1000, 250, 63, 16, and 4 µg/mL). After the cells were washed, they were treated with MTT solution and the absorption was assessed using an ELISA reader at 570 nm [43,44,45,70]. As a pharmacological reference drug, Celecoxib, a well-known anti-inflammatory agent, was utilized. As indicated in Figure 5, compounds **5a** and **7a** exhibited a considerably low cytotoxicity toward the WI-38 cell line which appeared to be dose-dependent. Compounds **5a** and **7a** exhibited a cytotoxicity activity (IC_50_ = 88.54 µg/mL, and 109.31 µg/mL, respectively) compared to that of the celecoxib reference drug (IC_50_ = 93.05 µg/mL). These findings are in accordance with previous studies which reported that substituted-2-thiohydantoin analogues possess a low cytotoxicity toward epithelial breast cells. Our results indicated that compounds **5a** and **7a** are promising antidiabetic agents with dual inhibitory activity and low cytotoxicity toward non-toxic human lung fibroblast cells [44,71]. 

### 2.4. Assessment of Antioxidant Activity

Reactive oxygen species (ROS) mediate several cellular processes by regulating the activity of enzymes involved in signal transduction [23]. Accumulation of ROS induces oxidative stress and toxic effects in cells that can cause damage to the cellular components and promote the development of diseases such as cancer, neurodegenerative diseases, and T2DM [25]. Accordingly, the discovery of antidiabetic compounds with potential antioxidant activity has been considered an appealing strategy for the management of T2DM. Encouraged by the inhibitory activity and cytotoxicity of compounds **5a** and **7a** and to gain insights into the mode of action, we assessed the antioxidant activity of the compounds by evaluating their potential to scavenge free radicals and to produce ROS.

#### 2.4.1. Evaluation of Free Radical Scavenging Activity

**Figure 6:**
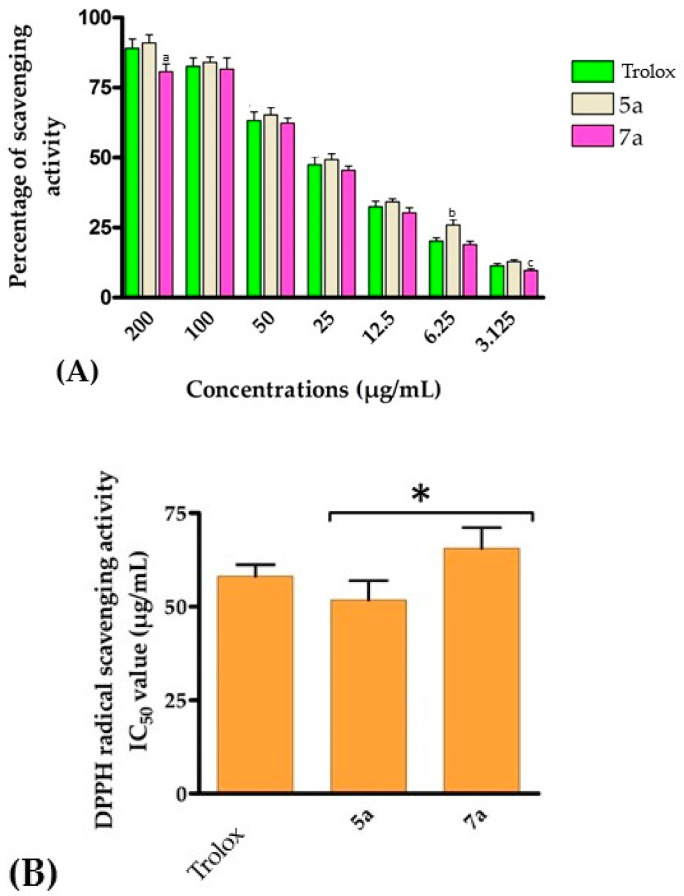
Free radical scavenging activity of compounds **5a**, **7a**, and Trolox toward DPPH substrate (n = 4). (**A**) Percentage of scavenging activity. (**B**) IC_50_ values (μg/mL). Data are presented as mean ± SD. a *p* < 0.05 versus Trolox at concentration 200 μg/mL, b *p* < 0.05 versus Trolox at concentration 6.25 μg/mL, c *p* < 0.05 versus Trolox at concentration 3.125 μg/mL,* *p* < 0.05 versus Trolox for the IC_50_ value.

The antioxidant capability of compounds **5a** and **7a** was estimated utilizing the DPPH (2,2-diphenyl-1- picrylhydrazyl) scavenging assay to assess the free radical scavenger activity. DPPH is a stable free radical substrate with a purple color which upon reaction with an antioxidant compound, converts into 1,1-diphenyl-2-picryl hydrazine, a colorless compound. The DPPH assay is widely applied for the investigation of superoxide radical scavenging activity [72,73]. In this regard, compounds **5a** and **7a** were incubated at different concentrations (3.12, 6.25, 12.5, 25, 50, 100, and 200 µg/mL) with DPPH substrate and the absorption was assessed by ELISA at 510 nm. In our investigations, Trolox, a water-soluble analogue of vitamin E, was applied as a standard antioxidant reference drug [74]. As indicated in Figure 6, compounds **5a** and **7a** demonstrated a significant and dose-dependent scavenging activity toward the free radical of the DPPH substrate. Interestingly, our findings revealed that compound **5a** (IC_50_ = 51.75 µg/mL) exhibited a potent free radical scavenging activity, compared to that of Trolox (IC_50_ = 58.09 µg/mL). On the other hand, compound **7a** showed a considerable free radical scavenging activity with IC_50_ of 65.5 µg/mL. These results are in agreement with previous reported studies which demonstrated that 2-thiohydantoins possess a potential antioxidant activity [32,41,75,76]. Recently, Camargo et al. reported the antioxidant activity of a set of C5-substitued-2-thiohydantoins and showed that this class of compounds possesses IC_50_ values of 40–200 µM toward DPPH-based scavenging activity [41]. Further, Qamar et al. synthesized and evaluated the antioxidant activity of some novel N3-substituted-2-thiohydantoins. The authors showed that this class of compounds possesses DPPH inhibition ranging from 2.77 to 47.98% at 100 µg/mL [39]. Our findings revealed that compounds **5a** and **7a** (1,3,5-trisubstitued-2-thiohydantoin) exhibited substantial antioxidant capability compared to that of the well-known antioxidant drug Torloxo. Antioxidant activity is a critical criterion for pharmacological bioactive compounds as it plays a crucial role in lessening the cellular toxicity associated with diseases [77]. Collectively, our results indicated that compounds **5a** and **7a** exhibited potential DPPH-based scavenging activity which could contribute to the enhancement of their antidiabetic effect and diminish the severity of the disease.

#### 2.4.2. Evaluation of Reactive Oxygen Species Production

**Figure 7:**
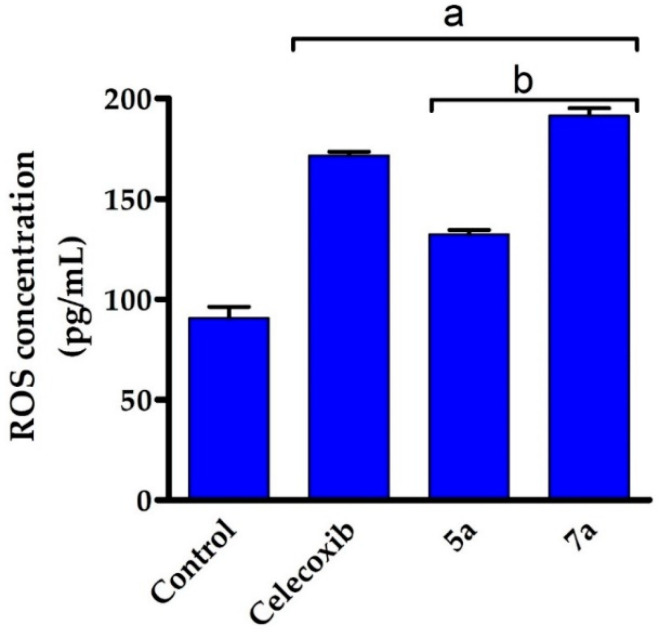
Effect of compounds **5a**, **7a**, and Celecoxib on the production of ROS (n = 4). Data are presented as mean ± SD. a *p* < 0.05 versus Control, b *p* < 0.05 versus Celecoxib.

The antioxidant metabolites and enzymes (such as NADPH oxidase, lipoxygenase, and cytochrome P450) act together to mediate the production of ROS and diminish the counteractive oxidative damage. ROS can cause damage to DNA, lipid, proteins, and nucleic acids and the destruction of cell components leading to cell death [24,78]. Toward this end, we aimed to investigate whether the mode of action of this class of compounds involved the generation of ROS. ROS-producing compounds upon reaction with a reducing agent in the presence of oxygen afford superoxide O^•−2^ radical and hydrogen peroxide (H_2_O_2_) which could be quantitively estimated [79,80]. Thus, compounds **5a** and **7a** (at 50 µM concentration) were incubated with the reducing agent dithiothreitol (DTT) and the generated H_2_O_2_ was subsequently assessed by monitoring the oxidation of phenol red in the presence of horseradish peroxidase (HRP) at the absorbance of 610 nm**.** We first examined the validity of the assay by examining the activity of a known ROS-producing standard drug. In our study, Celecoxib was utilized as a reference pharmacological ROS-generating drug [81,82]. As shown in Figure 7, celecoxib, in agreement with reported studies, demonstrated a substantial capability to produce ROS (171.6 pg/mL), compared to the control. Next, we evaluated the potency of compounds **5a** and **7a** to produce ROS at a 50 µM concentration. Our results showed that compound **7a** exhibited a similar potency as compared to Celecoxib to generate ROS (191.5 pg/mL). Interestingly, compound **5a** showed a low potential to produce ROS as indicated by monitoring the generated H_2_O_2_ (132.4 pg/mL), compared to the control experiment. These results indicated that compound **5a** possessed substantial antioxidant activity through its potent free radical scavenging activity and its low tendency to generate ROS. Our findings are in accordance with previous studies which demonstrated that 1,3-disubstituted-2-thiohydantoin induces upregulation in the levels of antioxidants in vivo including (CAT, SOD, and GSH) antioxidant activity [83]. 

Together, our results indicated that compounds **5a** and **7a** exhibited substantial antioxidant activity via the scavenging free radicals and the generation of ROS at low levels. These activities could be attributed to the electron-donating property and the exchangeable protons of the 2-thiohydantoin scaffold which effectively interact with the free radicals to afford stable molecules [41,84]. Therefore, this class of compounds could be applied to preserve the cells from oxidative stress while employing their antidiabetic effect [85]. It should be noticed that previous studies mainly investigated the antioxidant activity of 3N or C5-substituted-2-thionhydantoins [32,39,41,75,76]. To the best of our knowledge, this is the first report to explore the antioxidant activity of 1,3,5-trisubstituted-2-thiohydantoins. 

**Figure 8:**
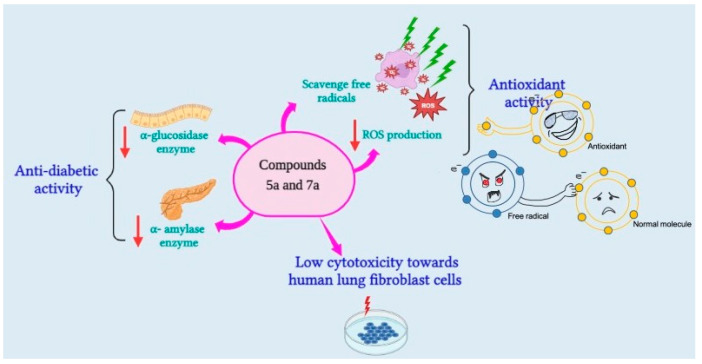
Illustration of the antidiabetic action of compounds **5a** and **7a**.

Based on these results, compounds **5a** and **7a** could be considered as lead 1,3,5-trisubstituted-2-thiohydantoin scaffolds that could be used for the development of potential antidiabetic compounds. Our findings revealed that compound **5a** and **7a** demonstrated effective antidiabetic activity via several mechanisms including the dual antagonist effect toward α-amylase and *α*-glucosidase activities, the low cytotoxicity toward normal epithelial cell line (WI-38), and the ability to scavenge free radicals, and to diminish the production of ROS (Figure 8). The potency and wide-range of antidiabetic effects of these compounds could be relevant to various diabetic-related diseases. Further investigations should be carried out to assess the bioactivity and antidiabetic activity of compounds **5a** and **7a** in animal models. 

### 2.5. Molecular Modelling Simulation Study

Molecular docking simulation has been successfully and extensively applied to examine the mode of interaction and binding score of a bioactive ligand toward the active site of a targeted protein [86,87,88,89,90,91,92]. In our study, we showed that the 1,3,5-trisubstituted-2-thiohydantoin scaffold possessed potential antidiabetic and antioxidant activity by targeting the activity of α-glucosidase and α-amylase enzymes. To gain more insights into the correlation between structural features of this class of compounds and the inhibitory activity of compounds, we performed extensive molecular modelling studies to explore the binding affinity and mode of binding of compounds **5a** and **7a** toward the α-amylase and α-glucosidase binding pockets. In the protein database, there are several X-ray structures available for α-amylase enzyme, while for the α-glucosidase enzyme only the 3D-structure of plant α-glucosidase has been reported. In our investigations, we selected the protein structures which exhibited a high resolution and low RMSD values; mainly α-glucosidase enzyme co-crystallized with acarbose (PDB code: *3w37*) and α-amylase enzyme co-crystallized with myricetin (PDB code: *4gqr*) [93,94]. Toward our aim, we initially assessed and verified the applied parameters of the docking protocol by affirming the binding conformations of the co-crystallized ligands toward α-glucosidase and α-amylase enzymes, as compared to the reported data. Subsequently, the verified protocol was employed to accomplish the molecular docking simulation of 1,3,5-trisubstituted-2-thiohydantoin compounds and the acquired data were analyzed to assess the best interacting conformers, affinity scores, and mode of interaction (H-bonding, hydrophobic, and arene–arene interactions). Table 1 explains the affinity scores and the different types of interactions of the best conformations into the active site of α-glucosidase and α-amylase cavities. The results demonstrated that compounds **5a** and **7a** possessed substantial affinity scores which revealed thermodynamic favorable interactions toward both α-glucosidase and α-amylase enzymes. Further, the most stable conformations (top-ranked) of compounds **5a** and **7a** indicated that these compounds adequately fitted into the pocket of α-glucosidase and α-amylase cavities and could form a network of hydrophobic and hydrophilic interactions with the main amino acid residues in the active sites. 

**Figure 9:**
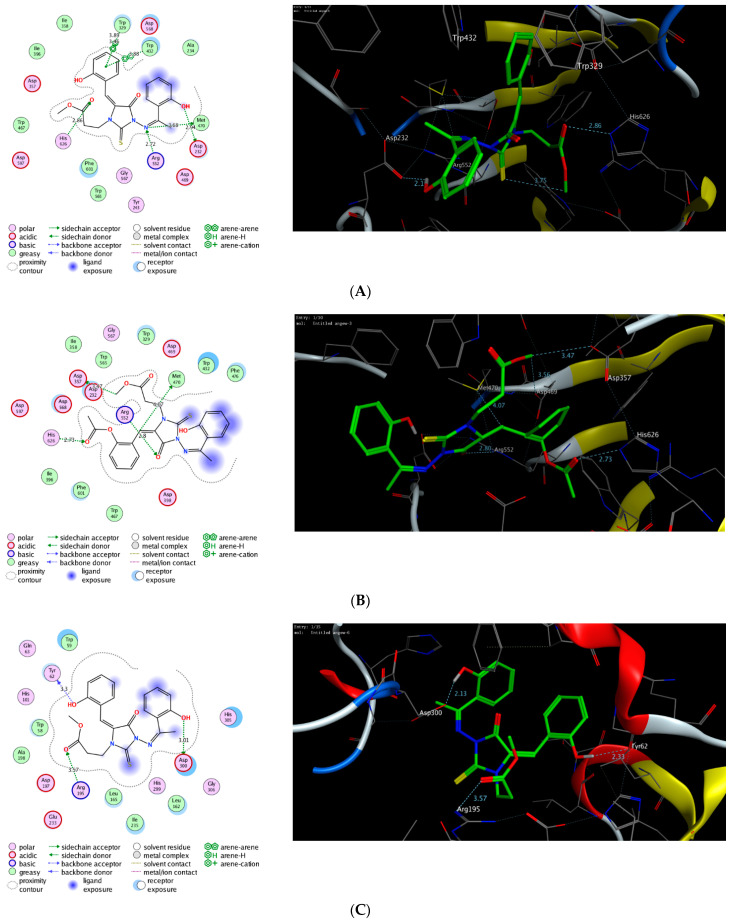

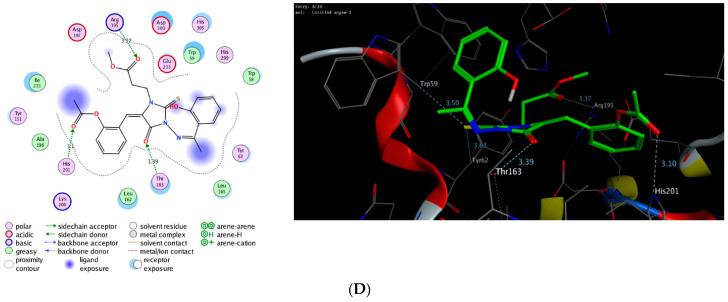
Interactive conformations (2D) and binding mode (3D) of compound 5a (**A**,**C**) and 7**a** (**B**,**D**) in the active cavity of α-glucosidase (PDB code: *3w37*) and α-amylase (PDB code: *4gqr*) enzymes.

Toward α-glucosidase enzyme, the 1,3,5-trisubstituted-thiohydantoin scaffold demonstrated a high binding energy through a network of interactions with the amino acid residues in the enzyme pocket. As illustrated in Figure 9, the most stable conformer of compound **5a** demonstrated the ability to form a set of H-bonding with several essential amino acid residues. The results revealed that the 2-(1-hydrazineylideneethyl)-phenol moiety at the N3 position bound through the phenolic-OH and the azomethine groups to three amino acid residues (Arg552, Met470, and Asp232). The 2-vinyl-phenol moiety at the C5 position also participated in the binding by interacting with two Trp residues via arene–arene interaction (Trp329 and Trp432). Further, the methyl-proponate moiety at the N1 position was able to form H-bonding with the His626 residue. The stability of the conformer was also stabilized by a network of hydrophobic interactions with greasy residues (Ala234, Ile358, Ile396, Trp467, Trp565, Phe601). These results indicated that the substitution at N1, N3, and C5-positions of thiohydantoin scaffold played a crucial rule in the binding affinity of this class of compounds toward the active site of the α-glucosidase receptor. Similarly, the conformer of compound **7a** demonstrated considerable interactions with the residues of the active pocket. As shown in Figure 9, the presence of the acetyl group in the scaffold resulted in a different mode of binding and configuration with lower binding energy (-11.42 kcal/mol), as compared to compound **5a** (-13.28 kcal/mol). The metyl-proponate moiety displayed the ability to bind to two Asp residues (Asp357 and Asp469). Although the 2-(hydrazineyl)-phenol moiety did not participate in the binding, the thiohydantoin scaffold demonstrated an H-bonding interaction with the Arg552 residue. The acetyl group played a considerable role in the binding affinity of the compound by forming H-bonding with the His626 residue. Together, these findings indicated that the lower inhibitory activity of compound **7a**, as compared to compound **5a**, could be attributed to the different mode of binding in which the 2-(hydrazineyl)-phenol moiety was directed far from the residues in the active site of the α-glucosidase pocket.

Regarding α-amylase enzyme, compounds **5a** and **7a** displayed a substantial affinity score toward the active site (−10.36 and -11.07 kcal/mol, respectively). Thus, the conformer of compound **5a** demonstrated the capability to interact with the residues in the active site through the three substituent- moieties at the N1, N3, and C5 positions. In this regard, the phenolic-OH at the N3- and C5- positions displayed the ability to form H-bonding with the Asp300 and Tyr62 residues, respectively. Further, the methyl-proponate at the N1 position formed H-bonding with Arg195 residue. The stability of this conformer was enhanced by the hydrophobic interactions with Trp58, Trp59, Leu162, Leu165, Ala198, and Ile235 residues. Like in the α-glucosidase-pose, the presence of acetyl group caused a shift in the binding mode of compound **7a**. As shown in Figure 9, the binding of compound **7a** into the active site was mainly based on the interaction of the methyl-proponate and hydrazineyl moieties with the Arg195 and Thr163 residues, respectively. In the most stable conformer, the 2-vinyl-phenol moiety was directed away from the active site and could not participate in the binding of the compound. However, the thiohydantoin scaffold directed to the binding site and could form two stable H-bonding through the thiocarbonyl group to Trp59 and Tyr62 residues. These observations could explain the lower inhibitory activity of compound **7a**, as compared to compound **5a**. Collectively, our study revealed the mode of the binding of this class of compounds toward the α-glucosidase and α-amylase pocket which was in harmony with the observed in vitro inhibitory activity. Further, our findings indicate that 1,3,5-trisubstitued-thiohydantoin could be considered as a lead scaffold for the development of potent antidiabetic agents.

## 3. Materials and Methods

### 3.1. Reagents and Instruments 

The chemicals and solvents in highly purified form (> 95%) were obtained from TCI, Alfa Aesar, or Sigma-Aldrich. Standard solvents used in the synthesis were purified by distillation following standard procedures. Thin layer chromatography was utilized to judge the reaction progress and to verify the product purity after purification. The TLC plates were acquired as silica gel plates pre-coated on aluminum sheets from Merk (Darmstadt, Germany). The structure of all synthesized compounds was characterized and elucidated by analytical techniques including elemental analysis, melting point, nuclear magnetic resonance, and FT-IR analysis. The elemental analysis was achieved using a Perkinelmer 2408 CHN analyzer. MEL-TEMP II instrument was utilized to perform the melting point assessment. The ^1^H- and ^13^C-NMR spectra were recorded at 300 MHz on a Buker spectrometer (DPX-400) for a solution of the sample in DMSO-*d6*. The coupling constant was calculated, and the chemical shift was expressed relative to the tetramethylsilane standard. Perkinelmer 337 spectrophotometer was utilized to acquire the IR spectra of KBr discs. The mass spectroscopic analysis was achieved using a mass spectrometer (Finnigan MATSSQ-7000) at 70 eV.

### 3.2. Compound Synthesis and Structure Characterization 

#### 3.2.1. Synthesis of 3-(2-hydroxyphenyl ethylidene) amino-2-thioxoimidazolidin-4-one (**3**) 

To a mixture of 2-hydroxy acetophenone thiosemicarbazone **2** (2.1 g, 10 mmol) in EtOH (50 mL) was added to fused sodium acetate (2.5 g, 30 mmol), and subsequently treated with ethyl chloroacetate (1.23 g, 10 mmol). The resultant mixture was refluxed and followed by TLC analysis. After the TLC analysis revealed a complete reaction (almost 16 h), the reaction was cooled to an ambient temperature and the resulting solid was separated by filtration. The obtained residue was washed with acetone, and dried to provide a crude yellow solid. Finally, the crude reside was recrystallized utilizing ethanol to afford compound **3** as yellow crystals. R*_f_*: 0.39 (DCM:MeOH 8:2, visualized with 1.3% Ninhydrin solution). Yield 86%, m.p. 225 °C. FT-IR (KBr)γ_max_: 3430 (OH), 3228 (NH), 1698 (C=O), 1628 (C=N), 1605, 1580 (C=C), 1121, 1036 (C-O) cm^−1^. ^1^H-NMR (DMSO-*d6*, 300 MHz, ppm): δ 2.52 (s, 3H, CH_3_), 4.03 (s, 2H, CH_2_ imidazole ring), 6.93–6.95 (m, 2H, Ar-H), 7.33–7.37 (t, 1H, Ar-H), 7.65–7.68 (d.d, 1H, Ar-H), 12.14 (br-s, 1H, OH), 12.59 (s, 1H, NH). ^13^C-NMR (DMSO-*d6*, 100 MHz, ppm): δ 174.05 (C=S), 166.44 (C=O), 163.57 (C-O), 159.52 (C=N), 132.25, 129.59, 119.56, 119.37, 117.50 (C-Aromatic), 33.99 (C-S of imidazole ring), 14.81 (CH_3_). Anal. Calcd for C_11_H_11_N O_2_ S (MWT = 49): C, 53.02; H, 4.43; N, 16.86. Found: C, 52.89; H, 4.27; N, 16.67.

#### 3.2.2. Synthesis of Compounds **4a**–**b**

Compound **3** (2.48 g, 10 mmol) was dissolved in dimethylformamide (25 mL) and subsequently treated with fused sodium acetate (2.5 g, 30 mmol) and aromatic aldehyde (mainly; 2-hydroxybenzaldehyde and 5-bromo-2-hydroxybenzaldehyde, 10 mmol). The obtained mixture was heated to reflux for 12–16 h (as indicated by TLC analysis), and then treated with ice-cold water to quench. The resultant mixture was subsequently neutralized with 1% aqu. HCl (pH~7) and was allowed to stand at 0 °C for 4 h, during which a solid precipitate started to form. The mixture was filtered and washed with water then cold ethanol to provide the crude residue. Purification of the resultant residue by crystallization using ethanol finally furnished compound **4** as yellow crystals. 

##### 5-(2-Hydroxybenzylidene)-3-(2-hydroxyphenylethylidene)-amino-2- thioxoimidazolidin-4-one (**4a**)

The entitled compound was synthesized staring from 2-hydroxybenzaldehyde to give compound **4a** as yellow crystals. R*_f_*: 0.42 (DCM:MeOH 9:1, visualized with 1.3% Ninhydrin solution). Yield 67 %, m.p. 256 °C. FT-IR (KBr)γ_max_: 3456–3380 (br. OH), 3222 (NH), 1685 (C=O), 1629 (C=N), 1613, 1592 (C=C), 1125, 1083, 1036 (C-O) cm^−1^. ^1^H-NMR (DMSO-*d6*, 300 MHz, ppm): δ 2.58 (s, 3H, CH_3_), 7.02–7.79 (m, 8H, Ar-H), 7.94 (s, 1H, H-olefinic), 10.51 (s, 1H, OH), 12.42 (s, 1H, OH), 12.70 (br-s, 1H, NH). ^13^C-NMR (DMSO-*d6*, 100 MHz, ppm): δ 174.03, 167.64, 167.53 * (C=O and C=S), 159.52, 159.40 * (C=N), 157.94, 157.51 * (C-O), 133.24, 132.55 *, 132.38, 132.24 *, 130.17, 129.85*, 129.59, 128.69 *, 125.83, 120.92, 120.77, 120.72 *, 120.14, 119.74 *, 117.50, 116.59 *, 15.29, 14.81 * (* refers to the *E*/*Z* isomer). Anal. Calcd. for C_18_H_16_N_3_O_3_S (MWT = 353): C, 61.19; H, 4.25; N, 11.90. Found: C, 61.01; H, 4.204; N, 11.68. Ms m/z (%): 353 (M^+^, unstable), 311 (7.63), 310 (36.02), 295 (1.94), 294 (4.03), 293 (19.72), 275 (1.10), 268 (3.50), 267 (2.23), 255 (1.05), 254 (9.70), 253 (55.38), 252 (20.19), 251 (100), 237 (1.20), 266 (2.03), 211 (2.10), 210 (11,95), 149 (3.56), 148 (8.70), 147 (1.80), 136 (2.57), 135 (8.54), 134 (40.58), 133 (41.86), 132 (3.72), 121 (1.49), 120 (11.65), 119 (13.19), 118 (1.36), 108 (2.64), 107 (3.67), 106 (3.43), 105 (4.15), 104 (3.42), 103 (1.04), 102 (1.8), 101 (1.81), 93 (5.60), 92 (6.63), 91 (36.23), 90 (2.92), 89 (2.79), 79 (2.64), 78 (2.75), 77 (5.38), 76 (1.28), 65 (24.27), 64 (5.22), 63 (5.99), 52 (1.18), 51 (2.59) (Figure S2b, Scheme S1).

##### 5-(5-Bromo-2-hydroxybenzylidene)-3-(2-hydroxyphenylethylidene)-amino-2-thioxoimidazolidin-4-ones (**4b**)

The entitled compound was synthesized staring from 5-bromo-2-hydroxybenzaldehyde to afford compound **4b** as yellow crystals. R*_f_*: 0.44 (DCM:MeOH 9:1, visualized with 1.3% Ninhydrin solution). Yield 78%, m.p. 263 °C. FT-IR (KBr)γ_max_: 3461–3360 (br. OH), 3227 (NH), 1691 (C=O), 1630 (C=N), 1607, 1586 (C=C), 1127, 1086, 1033 (C-O) cm^−1^. ^1^H-NMR (DMSO-*d6*, 300 MHz, ppm): δ 2.56 (s, 3H, CH_3_), 6.93–7.79 (m, 7H, Ar-H), 7.96 (s, 1H, H-olefinic), 10.84 (s, 1H, OH), 12.31 (s, 1H, OH), 12.74 (br-s, 1H, NH). ^13^C-NMR (DMSO-*d6*, 100 MHz, ppm): δ 174.00, 167.72, 167.35 (C=O and C=S), 162.77, 159.42 *, 157.42, 156.56 * (C-O), 138.91, 134.44, 132.53 *, 130.94, 129.81 *, 124.44, 123.08, 122.76 *, 120.33, 120.10 *, 119.70, 119.50 *, 118.62, 117.53 *, 111.41, 110.92 * (C-Aromatic and olefinic), 15.32, 14.77 * (CH_3_) (* refers to the *E*/*Z* isomer). Anal. Calcd. for C_18_H_14_BrN_3_O_3_S (MWT = 431): C, 50.12; H, 3.25; N, 9.74. Found: C, 50.03; H, 3.09; N, 9.51.

#### 3.2.3. General Procedure for the Synthesis of Methyl [5-arylidnene-4-oxo-3-(2-hydroxyphenyl ethylidene) amino-2-thioxoimidazolidin-1-yl] propionates (**5a**–**b**)

To a solution of compound **4** (10 mmol) in DMF (50 mL) was added methyl acrylate (0.87 g, 10 mmol) at 0 °C, followed by triethylamine (3 mL, 30 mmol). The resultant mixture was heated to reflux for 12–14 h and the progress of the reaction was followed by TLC analysis. After the TLC analysis demonstrated the consumption of all starting material, the mixture was cooled again to 0 °C and was treated with water to quench. The resulting mixture was treated with 1% HCl solution until pH optimized to pH~7. The obtained precipitate was separated, washed, and dried. Recrystallization of the resultant crude product utilizing ethanol successfully provided compounds **5a**–**b**. 

##### Methyl [5-(2-hydroxybenzylidene)-4-oxo-3-(2-hydroxyphenylethylidene) amino-2-thioxoimidazolidin-1-yl] propionate (**5a**)

The entitled compound was synthesized staring from compound **4a** to furnish compound **5a** as yellow crystals. R*_f_*: 0.42 (PE:EA 4:1, visualized with 1.3% Ninhydrin solution). Yield 65%, m.p. 189 °C. FT-IR (KBr)γ_max_: 3460–3390 (br. OH), 1753, 1691 (C=O), 1629 (C=N), 1613, 1583 (C=C), 1126, 1093, 1038 (C-O) cm^−1^. ^1^H-NMR (DMSO-*d6*, 300 MHz, ppm): δ 2.60 (s, 3H, CH_3_), 2.82–2.85 * (t, 2H, *J* = 6.1 Hz, CH_2_CO), 3.62 (s, 3H, OCH_3_), 4.16–4.19 * (t, 2H, *J* = 6.3 Hz, N-CH_2_), 6.94–7.03 (m, 5H, Ar-H), 7.31–7.43 (m, 3H, Ar-H), 7.69–7.71 * (m, 8H, Ar-H), 8.02 (s, 1H, H-olefinic), 10.57 (s, 1H, OH), 12.34 (s, 1H, OH). ^13^C-NMR (DMSO-*d6*, 100 MHz, ppm): δ 172.02, 171.51 *, 168.68, 167.69 *, 165.94 (2xC=O and C=S), 162.22, 160.06 * (C=N), 159.57, 159.43*, 157.57, 156.86 * (C-O), 132.71, 132.62 *, 132.44, 129.96, 129.73 *, 126.99, 120.65, 120.19*, 119.59, 119.49, 119.40 *, 117.70, 117.53 *, 116.63 (C-Aromatic and olefinic), 52.14 (OCH_3_), 39.34, 39.11 * (NCH_2_), 31.68, 31.54 * (CH_2_CO), 15.31, 14.82* (CH_3_) (* refers to the *E*/*Z* isomer). Anal. Calcd. for C_22_H_21_N_3_O_5_S (MWT = 439): C, 60.14; H, 4.78; N, 9.57. Found: C, 59.98; H, 4.44; N, 9.39. Ms m/z (%): 439 (M^+^, unstable), 355 (2.08), 350 (1.09), 348 (1.09), 341 (1.47), 338 (1.30), 336 (18.48), 337 (7.09), 336 (18.48),335 (100),334 (10.93), 333 (5.46), 332 (10.27), 331 (4.32), 327 (1.61), 321 (2.23), 320 (10.75), 319 (3.48), 3.18 (16.44) 317 (5.34), 316 (1.64), 315 (5.14), 305 (1.00), 304 (4.89), 303 (2.12), 301 (3.51), 300 (1.71), 298 (1.35), 290 (1.37), 283 (1.65), 282 (1.86), 281 (4.85), 278 (1.57), 277 (2.39), 276 (11.66), 271 (1.00), 270 (1.26), 269 (1.03), 262 (3.73), 261 (3.36), 260 (3.27), 253 (2.21), 252 (1.57), 250 (2.83), 249 (4.38), 248 (5.30), 236 (1.64), 253 (2.32), 234 (8.07), 233 (7.46), 232 (45.56), 228 (2.31), 221 (2.61), 220 (2.53), 219 (3.33), 218 (2.00), 208 (2.76), 207 (9.57), 206 (3.35), 205 (3.10), 202 (10.10), 201 (13.87), 200 (3.00), 199 (4.39), 192 (3.43), 191 (3.74), 189 (3.17), 188 (10.94), 187 (9.83), 186 (4.73), 182 (3.20), 177 (2.76), 176 (3.71), 175 (5.55), 174 (2.90), 173 (5.12), 172 (2.18), 171 (4.55), 162 (4.366), 161 (26.33), 160 (30.92), 158 (4.54), 149 (3.19), 148 (3.12), 147 (12.21), 146 (45.05), 145 (4.86), 143 (7.78), 141 (6.89), 136 (9.02), 135 (39.57), 134 (61.47), 133 (18.67), 132 (7.63), 121 (7.06), 120 (36.62), 119 (22.01), 118 (7.92), 117 (7.67), 106 (5.82), 105 (14.62), 104 (9.43), 103 (4.16), 102 (9.42), 101 (4.79), 95 (4.80), 94 (4.33), 93 (13.13), 92 (17.27), 91 (71.47), 90 (8.58), 87 (17.49), 85 (10.34), 78 (9.31), 77 (14.58), 76 (5.09), 75 (9.69), 65 (40.03), 64 (11.79), 63 (15.42), 59 (15.58), 55 (24.66),51 (8.14) (Figure S4b, Scheme S2).

##### Methyl [5-(5-Bromo-2-hydroxybenzylidene)-4-oxo-3-(2-hydroxyphenylethylidene) amino-2-thioxoimidazolidin-1-yl] propionate (**5b**)

The entitled compound was synthesized staring from compound **4b** to provide compound **5b** as yellow crystals. R*_f_*: 0.56 (PE:EA 3:2, visualized with 1.3% Ninhydrin solution). Yield 71 %, m.p. 192 °C. FT-IR (KBr)γ_max_: 3451–3395 (br. OH), 1756, 1689 (C=O), 1632 (C=N), 1615, 1591 (C=C), 1117, 1093, 1061 (C-O) cm^−1^. ^1^H-NMR (DMSO-*d6*, 300 MHz, ppm): δ 2.57, 2.59 * (s, 3H, CH_3_ of two isomer), 2.81–2.85 (t, 2H, *J* = 8 Hz, COCH_2_), 3.62 (s, 3H, OCH_3_), 4.15–4.18 (t, 2H, *J* = 6.2 Hz, NCH_2_) 6.93–7.69 * (m, 7H, Ar-H of two isomer), 7.78, 7.86 * (s, 1H, H-olefinic of two isomer), 10.85, 10.92 * (br-s, 1H, OH of two isomer), 12.23, 12.30 * (s, 1H, OH of two isomer). ^13^C-NMR (DMSO-*d6*, 100 MHz, ppm): δ 171.50, 168.92, 167.79, 167.35 *, 165.67 (2xC=O, C=S), 162.88, 159.41, 159.37 *, 156.57, 156.33 * (C-O), 134.70, 134.45 *, 132.74, 132.58 *, 131.10, 130.87 *, 129.92, 129.81 *, 125.59, 124.38 *, 123.04, 122.88, 122.70 *, 121.19, 119.66, 119.59 *, 118.65, 117.54 *, 110.94, 110.92 * (C-Aromatic and olefinic), 52.16 (OCH_3_), 39.19, 36.29 * (NCH_2_), 31.64, 31.26 * (CH_2_CO), 15.219, 14.76 * (* refers to the *E*/*Z* isomer). Anal. Calcd. for C_22_H_20_BrN_3_O_5_S (MWT = 517): C, 51.06; H, 3.87; N, 8.12. Found: C, 50.96; H, 3.63; N, 8.01.

#### 3.2.4. Synthesis of Compounds **6a**–**b**

A solution of compound **4** (10 mmol) in dry acetic anhydride (25 mL) was refluxed for 12–14 h and the reaction progress was observed by TLC analysis. The reaction was cooled to an ambient temperature and subsequently treated with ice-cold water. The resulting mixture was kept standing for 24 h in fridge, during which a precipitate was formed. The product was separated by filtration, washed with water, and dried under a high pressure vacuum. The resultant crude product was finally purified by recrystallization utilizing a suitable solvent (s) to furnish compounds **6a**–**b**. 

##### 5-(2-Acetoxybenzylidene)-3-(2-acetoxyphenylethylidene)amino-2-thioxoimidazolidin-4-one (**6a**)

The entitled compound was synthesized staring from compound **4a** to provide compound **6a** as pale-yellow crystals. R*_f_*: 0.53 (PE:EA 8:2, visualized with 1.3% Ninhydrin solution). Yield 62 %, m.p. 185 °C. FT-IR (KBr)γ_max_: 1758–1695 (br. C=O), 1629, (C=N), 1605, 1586 (C=C), 1125, 1096, 1036 (C-O) cm^−1^. ^1^H-NMR (DMSO-*d6*, 300 MHz, ppm): δ 2.03 (s, 3H, CH_3_), 2.28 (s, 3H, OCOCH_3_), 2.37 (s, 3H, NCOCH_3_), 7.13–7.85 (m, 9H, Ar-H and H-olefinic). ^13^C-NMR (DMSO-*d6*, 100 MHz, ppm): δ 169.54, 169.46 *, 169.07, 165.12 (C=O and C=S), 156.70 (C=N), 149.86, 149.74 *, 148.92, 148.35 *, 146.19 (C-O), 132.52, 132.17 *, 131.42, 131.02 *, 130.78, 130.04*, 129.98, 129.18 *, 128.69, 128.47 *, 127.92, 127.38, 127.19 *, 126.51, 126.09, 125.91 *, 124.85, 124.76 *, 124.31, 124.03 * (C-Aromatic), 81.09, 81.04 * (C-olefinic), 22.13, 21.51, 21.21 *, 21.11, 21.07 * (OCOCH_3_ and NCOCH_3_), 18.75 (CH_3_) (* refers to the *E*/*Z* isomer). Anal. Calcd. for C_24_H_21_N_3_O_6_S (MWT = 479): C, 60.12; H, 4.38; N, 8.77. Found: C, 5 = 60.05; H, 4.23; N, 8.51. Ms m/z (%): 479 (M^+^, unstable), 302 (1.04), 296 (1.78), 292 (1.44), 291 (1.25), 270 (2.16), 269 (19.35), 268 (100), 267 (2.08), 254 (15.71), 253 (83.24), 252 (8.26), 251 (40.56), 238 (1.28), 237 (5.32), 233 (1.06), 232 (5.66), 226 (2.91), 225 (1.35), 223 (1.09), 211 (3.37), 210 (18.70), 176 (1.16), 175 (7.63), 161 (2.56), 160 (1.07), 159 (1.39), 158 (1.09), 149 (7.48), 148 (2.42), 146 (1.03), 135 (14.41), 134 (45.11), 133 (30.14), 132 (4.68), 121 (4.01), 120 (25.81), 119 (15.08), 118 (2.83), 107 (6.90), 106 (6.41), 105 (5.81), 104 (5.58), 103 (1.66), 102 (3.73), 101 (2.08), 94 (1.72), 93 (8.45), 92 (10.68), 91 (49.87), 90 (4.94), 89 (4.85), 78 (4.58), 77 (9.53), 76 (2.36), 75 (3.18), 65 (42.61), 64 (9.12), 63 (10.58), 53 (3.43), 51 (5.42) (Figure S6a, Scheme S1).

##### 5-(5-Bromo-2-hydroxybenzylidene)-3-(2-acetoxyphenylethylidene) amino-2-thioxo-1-acetyl-imidazolidin-4-one (**6b**)

The entitled compound was synthesized staring from compound **4b** to furnish compound **6b** as pale-yellow crystals. R*_f_*: 0.47 (PE:EA 9:1, visualized with 1.3% Ninhydrin solution). Yield 69 %, m.p. 191 °C. FT-IR (KBr)γ_max_: 1751–1693 (br. C=O), 629 (C=N), 1610, 1586 (C=C), 1127, 1068, 1033 (C-O) cm^−1^. ^1^H-NMR (DMSO-*d6*, 300 MHz, ppm): δ 2.03 (s, 3H, CH_3_), 2.88 (NCOCH_3_), 2.32 (2× OCOCH_3_), 7.13–7.85 (m, 8H, Ar-H and H –olefinic). ^13^C-NMR (DMSO-*d6*, 100 MHz, ppm): δ 169.22, 169.15, 165.22 (C=O), 156.39 (C=S), 148.89, 148.56 (C-O), 145.82, 134.57, 131.08, 130.03, 129.83, 129.06, 128.40, 126.50, 126.14, 124.76, 124.01, 119.30 (C of Aromatic), 81.11 (C-olefinic), 22.10, 21.22, 21.19, 21.07 (4 ×CH_3_). Anal. Calcd. for C_24_H_20_BrN_3_O_6_S (MWT = 557): C, 51.71; H, 3.59; N, 7.54. Found: C, 51.63; H, 3.33; N, 7.37.

#### 3.2.5. Synthesis of Compounds **7a**–**b**

Compounds **7a**–**b** were synthesized following the protocol employed for the synthesis of compounds **6a**–**b**. Thus, compound **5a**–**b** (10 mmol) was dissolved in acetic anhydride (45 mL) and the obtained solution was heated to reflux under stirring for 12–16h. After the staring material was completely consumed, the reaction mixture was cooled to 0 °C. The mixture was subsequently transferred to ice-water and the obtained mixture was kept standing for 14 h at 0 °C. The mixture was filtered, and the collected crystals were washed and dried. Finally, the product was subjected to purification by recrystallization in a proper solvent (s) to provide compound **7a**–**b**. 

##### Methyl [5-(2-acetoxybenzylidene)-4-oxo-3-(2-hydroxyphenylethylidene) amino-2-thioxoimidazolidin-1-yl] propionate (**7a**)

The entitled compound was synthesized staring from compound **5a** to provide compound **7a** as pale-yellow crystals. R*_f_*: 0.48 (PE:EA 9:1, visualized with 1.3% Ninhydrin solution). Yield 61 %, m.p. 163 °C. FT-IR (KBr)γ_max_: 3406 (OH), 1749–1691 (br. C=O), 1631 (C=N), 1608, 1586 (C=C), 1125, 1083, 1061 (C-O) cm^−1^. ^1^H-NMR (DMSO-*d6*, 300 MHz, ppm): δ 2.38 (s, 3H, CH_3_), 2.62 (s, 3H, OCOCH_3_), 2.84–2.87 (t, 2H, *J* = 6 Hz, COCH_2_), 3.62 (s, 3H, OCH_3_), 4.17–4.21 (t, 2H, *J* = 8 Hz, NCH_2_), 6.96–7.73 (m, 9H, Ar-H and H-olefinic), 12.18 (s, 1H, OH). ^13^C-NMR (DMSO-*d6*, 100 MHz, ppm): δ 171.49 (C=S), 169.48, 169.22, 165.38 (3xC=O), 159.33 (C=N), 156.20 (C-O), 149.87, 132.85, 132.02, 130.09, 128.76, 127.36, 126.61, 124.39, 124.23, 123.87, 119.83, 119.67, 117.54 (C-Aromatic and olefinic), 52.16 (OCH_3_), 40.60 (NCH_2_), 31.61 (CH_2_CO), 21.13 (OCOCH_3_), 15.58 (CH_3_). Anal. Calcd. for C_24_H_23_N_3_O_6_S (MWT = 481): C, 59.87; H, 4.78; N, 8.73. Found: C, 59.66; H, 4.54; N, 8.66. Ms m/z (%): 481 (M^+^, unstable), 337 (10.25), 336 (16.83), 335 (100), 319 (13.20), 318 (13.63), 303 (6.21), 277 (1.03), 276 (15.30), 250 (2.25), 249 (3.63), 248 (5.07), 247 (1.00), 246 (1.44), 242 (1.18), 235 (1.73), 234 (7.13), 233 (6.73), 232 (43.04), 228 (1.96), 219 (2.23), 218 (2.17), 216 (2.42), 204 (4.66), 203 (7.42), 202 (39.27), 201 (11.72), 192 (2.52), 191 (2.10), 188 (10.05), 187 (8.11), 186 (4.27), 177 (3.13), 176 (4.07), 175 (14.95), 173 (4.23), 171 (2.90), 162 (2.32), 161 (12.11), 160 (29.25), 159 (21.45), 158 (4.38), 149 (2.39), 148 (2.48), 147 (10.56), 146 (47.78), 141 (6.24), 135 (30.97), 134 (55.84), 133 (16.01), 132 (6.55), 121 (6.38), 120 (25.74), 119 (16.83), 118 (3.61), 107 (6.32), 105 (9.74), 104 (7.49), 103 (2.00), 102 (8.15), 101 (4.13), 93 (6.15), 92 (13.06), 91 (60.36), 90 (5.58), 89 (9.11), 87 (15.59), 85 (7.69), 78 (5.67), 77 (9.63), 76 (3.91), 75 (3.47), 74 (3.85), 65 (30.53), 64 (9.18), 63 (10.79), 55 (17.52), 53 (3.37), 51 (5.87). (Figure S8b, Scheme S2).

##### Methyl [5-(5-bromo-2-acetoxybenzylidene)-4-oxo-3-(2-hydroxyphenylethylidene) amino-2-thioxoimidazolidin-1-yl] propionate (**7b**)

The entitled compound was synthesized staring from compound **5a** to give compound **7b** as pale-yellow crystals. R*_f_*: 0.61 (PE:EA 8:2, visualized with 1.3% Ninhydrin solution). Yield 73 %, m.p. 172 °C. FT-IR (KBr)γ_max_: 3417 (OH), 1750–1692 (br. C=O), 1628 (C=N), 1610, 1591 (C=C), 1128, 1093, 1061 (C-O) cm^−1^. ^1^H-NMR (DMSO-*d6*, 300 MHz, ppm): δ 2.28 (s, 3H, CH_3_), 2.32 (s, 3H, CH_3_), 2.36 (s, 3H, CH_3_), 2.61 (s, 3H, OCOCH_3_), 2.83–2.87 (t, 2H, CH_2_CO), 3.62 (s, 3H, OCH_3_), 4.17–4.20 (t, 2H, NCH_2_), 6.95–7.76 (m, 8H, Ar-H of two isomers), 12.10 (s, 1H, OH). ^13^C-NMR (DMSO-*d6*, 100 MHz, ppm): δ 171.50, 169.54, 169.48 *, 169.25, 169.16 *, 168.60, 165.24, 165.12 * (C=O and C=S of two isomers), 159.12, 156.39, 155.77 (C-O), 148.89, 148.83 *, 148.72, 148.56 * (C=N), 134.57, 134.40 *, 132.93, 131.27, 131.09, 130.95 *, 130.08, 130.02 *, 129.79, 129.02, 128.38, 126.49, 126.41 *, 126.27, 126.15 *, 125.61, 124.61, 124.16, 124.00 *, 119.72, 119.30, 119.20*, 117.59, (C-aromatic of two isomers), 81.11 (C-olefinic), 52.19 (OCH_3_), 40.46 (NCH_2_), 31.59 (CH_2_CO), 22.08, 21.59 *, 21.21, 21.10 (OCOCH_3_), 18.75, 15.58 * (* refers to the *E*/*Z* isomer). Anal. Calcd. for C_24_H_22_BrN_3_O_6_S (MWT = 559): C, 51.52; H, 3.93; N, 7.51. Found: C, 51.29; H, 3.63; N, 7.27.

### 3.3. Evaluation of α-Glucosidase Activity

An ELISA reader was used to assess the inhibition of α-glucosidase by 1,3,5-trisubstituted-2-thioxoimidazolidin-4-ones derivatives at various doses (1000, 100, 10, and 1 µg/mL) according to Peytam et al. [10]. The α-glucosidase enzyme (EC3.2.1.20, Saccharomyces cerevisiae (20 U/mg), Acarbose, and substrate (*p*-nitrophenyl-β-D-glucopyranoside) were provided by Sigma-Aldrich. Other chemicals were of the highest purity grade and purchased from Merck India. Enzyme was prepared in a potassium phosphate buffer (pH 6.8, 50 mM). Compounds **4a**–**b**, **5a**–**b**, **7a**–**b**, and Acarbose were dissolved in DMSO (10% final conc.). The different concentrations of the synthesized derivatives/Acarbose (20 µL), enzyme solution (20 µL), and potassium phosphate buffer (135 µL), were added in the 96-well plate and incubated at 37 °C for 10 min. Then, the substrate (25 µL, 4 mM) was added to the mentioned mixture and allowed to incubate at 37 °C for 20 min. Finally, the change in absorbance at 405 nm was monitored using a Cytation 3 hybrid microplate reader (BioTek, Winooski, VT, USA). The control and standard agents were DMSO and acarbose, respectively. GraphPad prism 6.0 was used to calculate the IC_50_ values of the investigated compounds from the nonlinear regression curve (Systat Software, Inc. Tools for Science) (https://www.graphpad.com/scientific-software/prism/ (accessed on 1 September 2022)). 

### 3.4. Evaluation of α-Amylase Activity

The inhibitory activity of synthesized 1,3,5-trisubstituted-2-thioxoimidazolidin-4-ones derivatives toward α-amylase activity was determined by ELISA analysis according to Bernfeld and Colowick [95]. The α-amylase enzyme was provided by Sigma-Aldrich and the stock solution was prepared in DMSO (10 mg/10 mL). The inhibitory activity of synthesized compounds **4a**–**b**, **5a**–**b**, **7a**–**b**, and Acarbose (reference drug) was determined at different concentrations (0.1, 1, 10, and 100 µg/mL). Briefly, α-amylase enzyme (100 mg, 1 unit per mL) was mixed in 100 mL of a 20 mM sodium phosphate buffer (pH 6.9). A solution of 3,5-dinitrosalicylic acid (DNSA, 96 mM) in 2 M NaOH (8 mL) was mixed with sodium potassium tartrate (5.31 mM), and deionized water (12 mL). The stock solutions of compounds **4a**–**b**, **5a**–**b**, **7a**–**b**, and Acarbose were prepared in DMSO (10% final conc.). Subsequently, 100 μL of synthesized derivatives/Acarbose solution and 100 μL of enzyme solution were mixed in vials and incubated at 25 °C for 30 min. After incubation, 100 μL of the DNSA reagent was added to this mixture, and then heated in a water bath at 85 °C for 15 min. The reaction mixture was taken from the water bath, cooled, and the absorbance value at 595 nm was assessed. Individual blanks were created in order to adjust the background absorbance. A control experiment was carried out following the same protocol using DMSO. The α-amylase enzyme activity and percentage of inhibition were determined using the following formula:

$$\%\;\mathrm{Inhibition}=\frac{control-test}{control}\times 100$$

### 3.5. Assessment of Cytotoxicity against the WI-38 Cell Line Using MTT Assay

The cytotoxicity of compounds **5a** and **7a** was assessed toward the human lung fibroblast WI-38 cell line (Sigma-Aldrich, St. Louis, MO, USA, product number 90020107) using MTT assay. The MTT test assay was performed in accordance with the previously stated procedure [96]. MTT (3-(4,5-dimethylthiazol-2-yl)-2,5-diphenyl tetrazolium bromide; Sigma catalog no. M2128) was dissolved in PBS at 5 mg/mL and processed to eliminate the insoluble residues. Briefly, cells (10^5^ cells/well) were treated for 48 h at 37 °C with compounds **5a** and **7a** at varied doses (1000, 250, 63, 16, and 4 µg/mL, DMSO stock solutions) in a 5% CO_2_ atmosphere. In our assessment, celecoxib, an inhibitor of cyclooxygenase 2, was employed as a standard reference drug. Following cell washing, MTT solution (180 µL/well) was applied to the cells and incubated at 37 °C for 4 h with shaking, and the absorbance was measured using an ELISA reader at 570 nm. 

### 3.6. Assessment of Free Radical DPPH Scavenging Activity 

According to Valko et al. [97], the ELISA approach was used to analyze the free-radical DPPH scavenging activity of compounds (**5a** and **7a**) at different concentrations (3.12, 6.25, 12.5, 25, 50, 100, and 200 µg/mL). The DPPH assay assesses the hydrogen-donating activity and thus provides an evaluation of the antioxidant activity via estimating the DPPH free-radical scavenging activity. The color intensity of the reaction mixture was measured after a purple-colored stable free radical (DPPH) was reduced into the yellow-colored diphenylpicryl hydrazinem. Briefly, 200 µL of different concentrations of compounds **5a** and **7a** were mixed with 50 µL of DPPH (0.659 mM) solution, and the mixture was incubated at 25 °C for 20 min. The absorbance of the reaction mixture was estimated after incubation at 510 nm. In our investigations, trolox was employed as standard reference drug [97]. A control reaction was performed, and the inhibitory efficiency of DPPH radical-scavenging activity was determined using the equation below:

% Inhibition = (A_0_ − A_1_)/A_0_ × 100 (1)

where A_0_ is absorbance of torolox and A_1_ is the absorbance of the compound.

### 3.7. Assessment of ROS-Generation Capability 

According to Nasiri et al., the antioxidant activity of compounds (**5a**, **7a**) and Celecoxib was measured by an evaluation of ROS-generation using ELISA analysis [98]. Three different solutions were prepared for the in vitro assessment of ROS-generation: phenol red, sodium hydroxide solution, and DTT. All reagents were provided by Sigma-Aldrich. A stock solution of DTT (20 mM) was prepared by dissolving 15.43 mg of DTT in 500 µL of Hank’s Balanced Salt Solution (HBSS), and the obtained solution was preserved at −20 °C. The stock solution was further diluted with HBSS to 0.8 mM for each measurement. The HRP-PR solution was prepared by dissolving 1.8 mg of horseradish peroxidase (HRP) and 3 mg of phenol red (PR) in 10 mL of HBSS. The obtained solution was kept at −20 °C and applied to each measurement. The experiment was performed using a 70 µL sample volume (20 µL compound, 20 µL DTT, 20 µL HRP-PR, 10 µL NaOH) on a transparent 384 well plate. The desired concentrations of compounds **5a** and **7a** were obtained by dissolving in DMSO with 0.01% TritonX100 and diluted with HBSS until the desired concentration was reached. Briefly, the compounds were initially incubated with DTT for 15 min. Subsequently, HRP-PR was added and incubated for additional 5 min. After incubation, 1N NaOH was applied, and the absorbance at 610 nm was measured using a plate reader spectrometer. The well plate was placed on a shaking plate during all of the incubation period and measurements. 

### 3.8. Molecular Modelling Simulation Study

Molecular modelling simulation is a potential computational analysis which can be applied to assess the binding energy and interaction of antagonist toward the binding site of a targeted receptor. In the current study, detailed in silico molecular docking simulation studies were performed to obtain insights into the binding mode and structural features of 1,3,5-trisubstituted-2-thiohydantoin (compounds **5a** and **7a**) conjugate with the active pocket of α-amylase and α-glucosidase enzymes. The molecular modelling studies were accomplished following our standard reported protocol utilizing the MOE program [86,87,88,89,90,91,92]. Fortunately, several crystal structures are available for both α-amylase and α-glucosidase enzymes. We selected the crystal structure of α-glucosidase enzyme co-crystallized with acarbose (PDB code: *3w37*) and the crystal structure of α-amylase enzyme which is co-crystallized with myricetin (PDB code: *4gqr*) [93,94]. The PDB formats of selected X-ray structures were obtained from the protein database (accessed on 1 August 2022). The 2D and 3D structures of ligands (**5a** and **7a**) were acquired from the Chemdraw builder program and MOE software, respectively. The obtained 3D structures of ligands were protonated and subsequently subjected to energy minimization utilizing the default mode of MOE program (forcefield Amber10:EHT: R-Field 1:80; Cutoff 8, 10, under rigid water molecules, and gradient 0.1 RMS kcal/mol). Similarly, the 3D structures of α-amylase and α-glucosidase enzymes were protonated, partial charges were expressed, and extra chains and water molecules were deleted. The MMFF94X force field was utilized to express the charge with energy minimization. The molecular docking process was performed using the Triangle Matcher placement method and the scoring was assessed using the London dG scoring function. The applied docking protocol was initially verified by ensuring that the co-crystallized ligand can form similar binding conformations and interactions with main amino acid residues as reported. The verified protocol was subsequently utilized to assess the mode of binding of antagonists (**5a** and **7a**) into the binding site of α-amylase and α-glucosidase enzymes. The acquired conformations (30 poses) were evaluated and the interactions with amino acid residues were analyzed, and binding energy was assessed. 

### 3.9. Statistical Studies

All investigations were applied in quadruplicate. Our data are presented as the mean standard error of the mean. The data were analyzed using one-way ANOVA, which was complemented by the Bonferroni post hoc test. The *p* value was used to determine the significance of the data; *p* > 0.05 was deemed non-significant, whereas *p* < 0.05 was considered to be significant. Statistical analysis was performed using the GraphPad prism program (Inc., San Diego, CA, USA, 2007).

## 4. Conclusions

In the current work, we designed, synthesized, and characterized novel 1,3,5-trisubstituted-2-thiohydantoin analogues. The structure of the synthesized compounds was extensively explored and elucidated by several analytical techniques including FT-IR, elemental analysis, and NMR. The antidiabetic potential of synthesized compounds was assessed by evaluating their inhibitory activity toward α-amylase and α-glucosidase enzymes. Our results revealed that the synthesized compounds displayed a substantial activity against both α-amylase and α-glucosidase enzymes. Among the assessed compounds, compound **5a** was recognized as the most potent analogue with significant antidiabetic activity as compared to the drug Acarbose. Exploration of the antidiabetic potential of compound **5a** demonstrated that this compound exhibited considerable cytotoxic activity toward WI-38 cells and substantial scavenging activity toward DPPH free radicals (IC_50_ = 51.75 µg/mL) with low potential to produce ROS. Further, in silico molecular modelling simulations revealed that this class of compounds possessed a significant binding score toward the active sites of α-amylase and α-glucosidase enzymes. Taken together, this study explored for the first time the structural features of trisubstituted-2-thiohydantoins to develop potent antidiabetic agents and affirmed that this class of compounds could be utilized as a lead structure to design compounds with antidiabetic and antioxidant potentials.

## Figures and Tables

**Table 1:** Binding energies and predicted interactions of conformers of compounds **5a** and **7a** toward the pocket of α-glucosidase (PDB: *3w37*) and α-amylase (PDB: *4gqr*) enzymes.

Protein (PDB Code)	Compound	S (kcal/mol)	Hydrophilic Interactions	Distance (A)	Hydrophobic Interactions
**α-glucosidase (*3w37*)**	**5a**	−13.28	Asp232 Trp329 Trp432 Met470 Arg552 His626	2.94 3.46 3.88 3.68 2.72 2.85	Ala234, Ile358, Ile396, Trp467, Trp565, Phe601
	**7a**	−11.42	Asp357 Asp469 Met470 Arg552 His626	3.47 3.56 4.07 2.80 2.73	Trp329, Ile358, Ile396, Trp432, Trp467, Met470, Phe476, Trp565, Phe601
**α-amylase (*4gqr*)**	**5a**	−10.34	Tyr62 Arg195 Asp300	2.33 3.57 2.13	Trp58, Trp59, Leu162, Leu165, Ala198, Ile235
	**7a**	−11.69	Trp59 Tyr62 Thr163 Arg195 His201	3.50 3.63 3.39 3.37 3.10	Trp58, Trp59, Leu162, Leu165, Ala198, Ile235

## Data Availability

Data is contained within the article and supplementary material.

## Supplementary Materials

The following supporting information can be downloaded at: https://www.mdpi.com/article/10.3390/ph15121576/s1. Figure S1: ^1^H NMR and ^13^C NMR spectra of compound **3**; Figure S2: ^1^H NMR, ^13^C NMR and mass spectra of compound **4a**; Figure S3: ^1^H NMR and ^13^C NMR spectra of compound **4b**; Figure S4: ^1^H NMR, ^13^C NMR, and mass spectra of compound 5a; Figure S5: ^1^H NMR and ^13^C NMR spectra of compound **5b**; Figure S6: ^1^H NMR, ^13^C NMR, and mass spectra of compound **6a**; Figure S7: ^1^H NMR and ^13^C NMR spectra of compound **6b**; Figure S8: ^1^H NMR, ^13^C NMR, and mass spectra of compound **7a**; Figure S9: ^1^H NMR and ^13^C NMR spectra of compound **7b**; Scheme S1: The represented mass fragmentation pattern of compounds **4a** and **6a**; Scheme S2: The represented mass fragmentation pattern of compounds **4a** and **6a**; Table S1: Inhibitory activities of 1,3,5-trisubstituted-2-thioxoimidazolidin-4-ones compounds toward α-glucosidase activity; Table S2: Inhibitory activities of 1,3,5-trisubstituted-2-thioxoimidazolidin-4-ones derivatives toward α-amylase activity; Table S3: In vitro cytotoxicity of compounds (**5a** and **7a**) against WI-38 cells; Table S4: Effect of Compounds (**5a** and **7a**) on scavenging DPPH free radical; Table S5: Effect of Compounds (**5a** and **7a**) on ROS generation.

## Sample Availability

Samples of the compounds **2**, **3**, **4a**, **4b**, **5a**, **5b**, **6a**, **6b**, **7a**, and **7b** are available from the authors.
