# Supplementary material for: Antidiabetic Potential of Novel 1,3,5-Trisubstituted-2-Thioxoimidazloidin-4-One Analogues: Insights into α-Glucosidase, α-Amylase, and Antioxidant Activities
Source: Pharmaceuticals (Basel). 2022 Dec 17;15(12):1576. doi: 10.3390/ph15121576 (PMC9785777; doi:10.3390/ph15121576)
Supplement: Supplementary file 1 [file pharmaceuticals-15-01576-s001.zip › pharmaceuticals-2050984-supplementary.pdf]

Supplementary Materials

Anti-diabetic Potential of Novel 1,3,5-Trisubstituted-2-Thioxoimidazloidin-4-one Analogues: Insights into α-Glucosidase, α-Amylase, and Antioxidant Activities

Salma M. Khirallah [1], Heba M. M. Ramadan [2], Hossam Aladl Aladl Aladl [3], Najla O Ayaz [4], Lina A.F Kurdi [5], Mariusz Jaremko [6], Samar Zuhair Alshawwa [7], and Essa M. Saied [8,9,*]

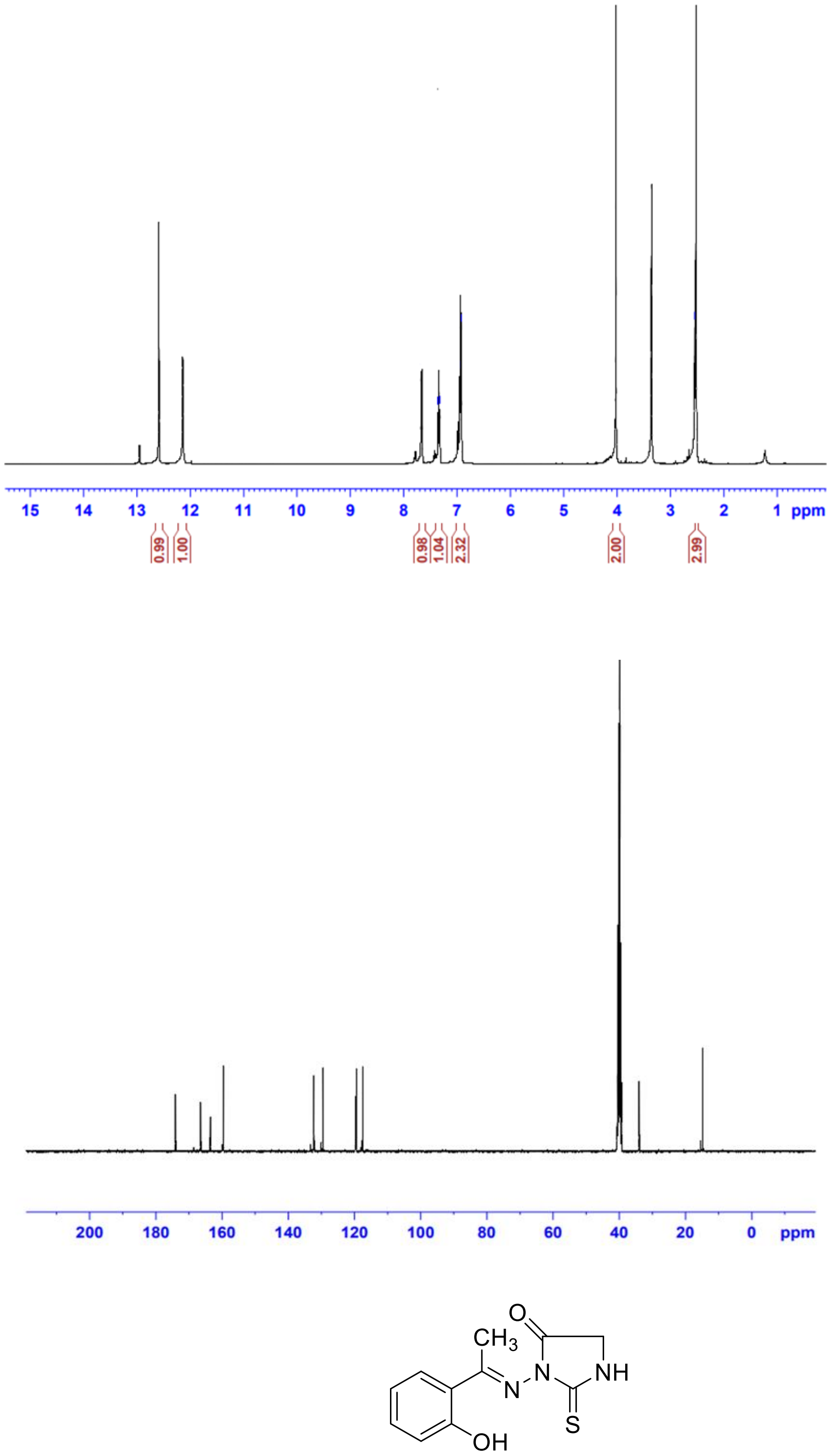

Figure S1. ^{1}H NMR and ^{13}C NMR spectra of compound 3

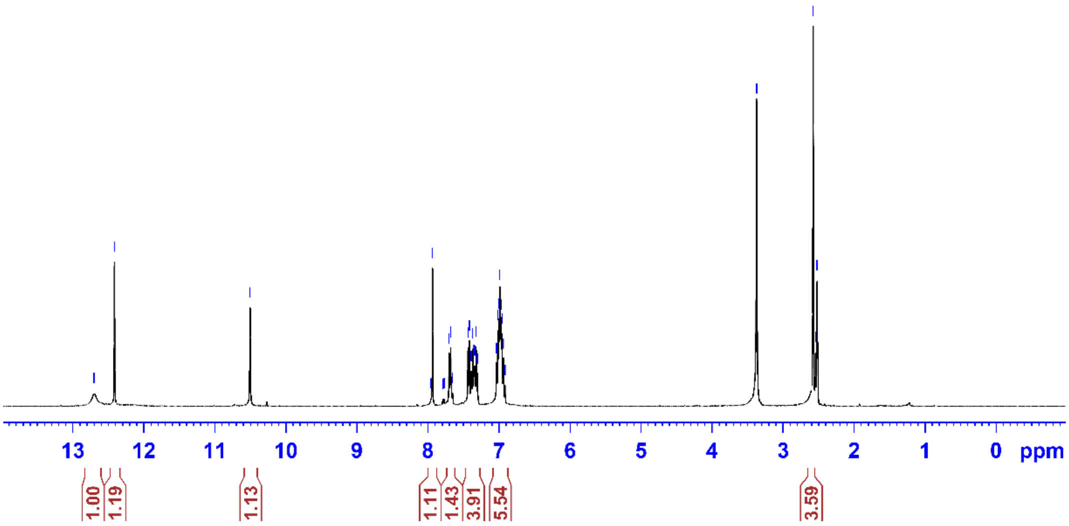

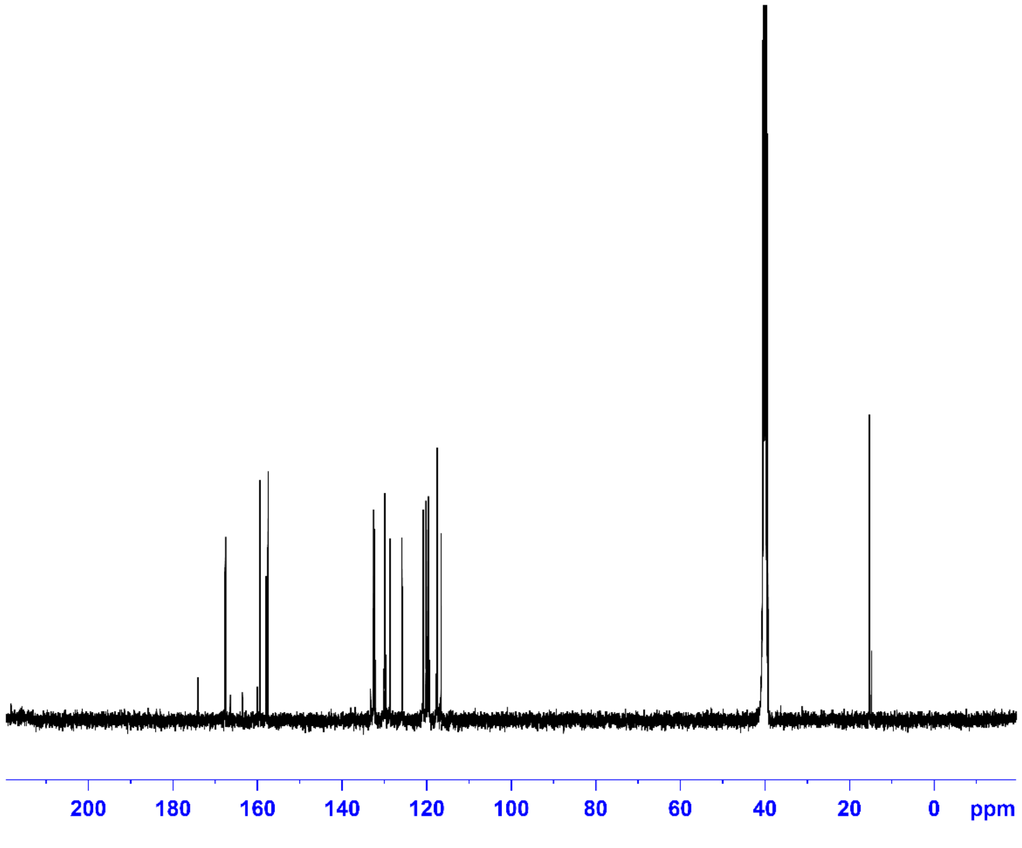

Figure S2a. ^{1}H **NMR and** ^{13}C **NMR spectra of compound 4a**

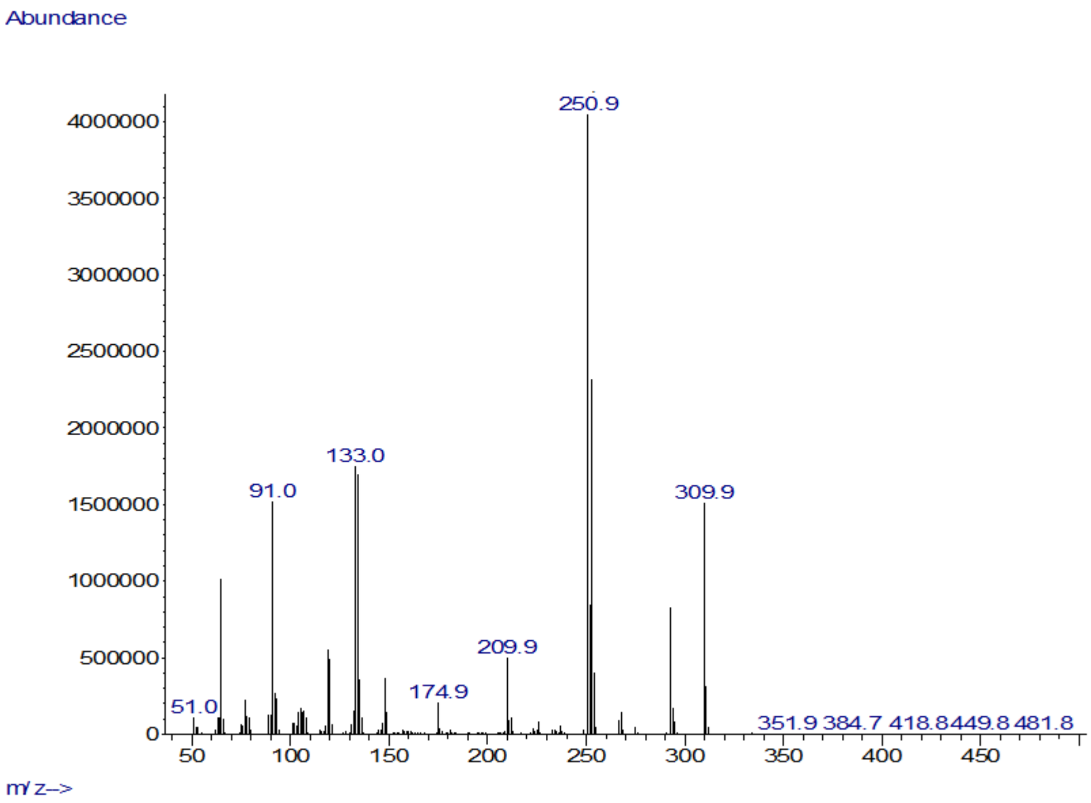

Figure S2b. Mass spectrum of compound 4a

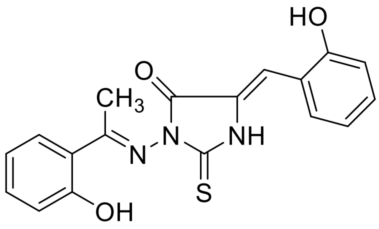

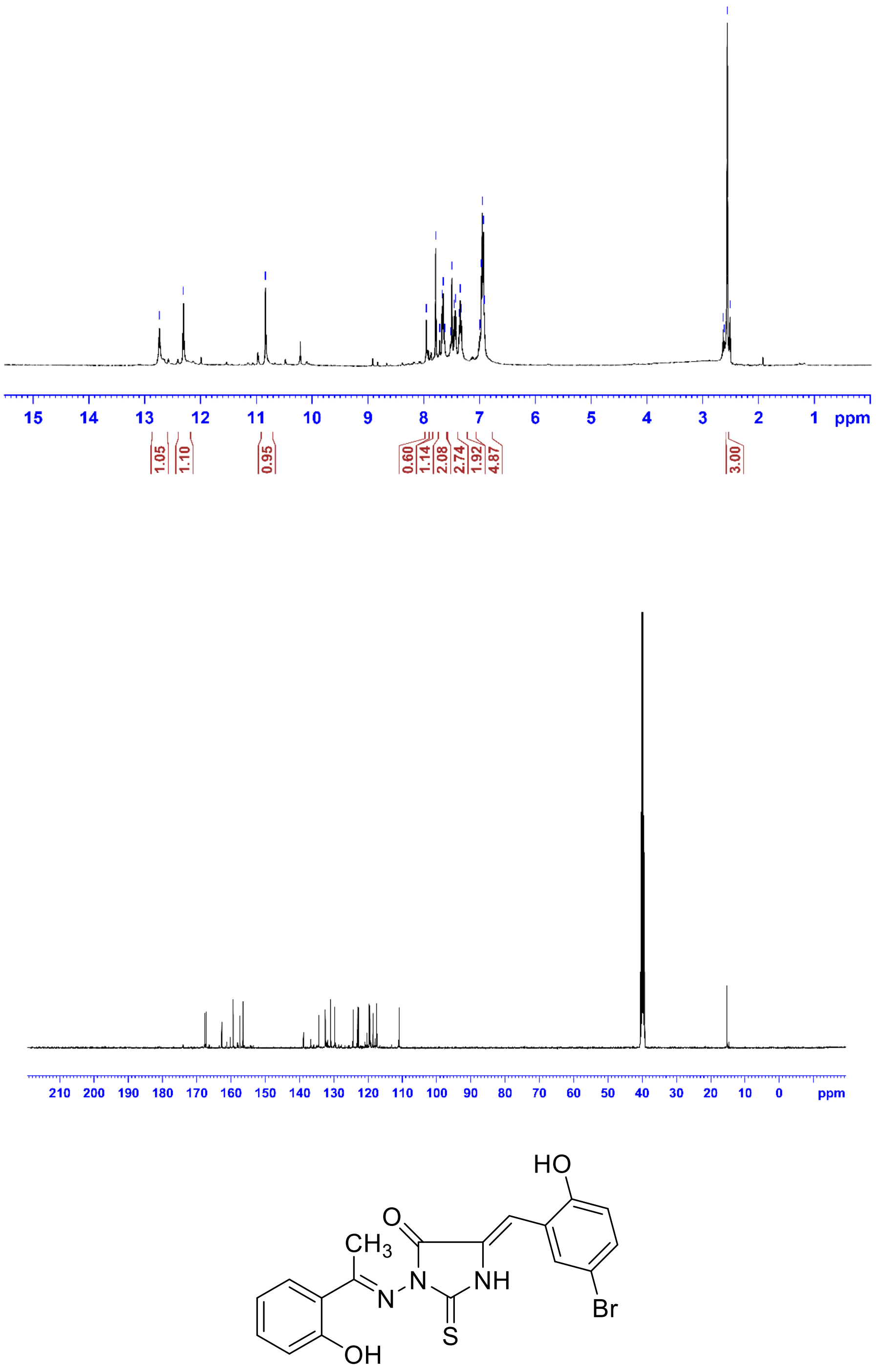

Figure S3. **^{1}H NMR and ^{13}C NMR spectra of compound 4b**

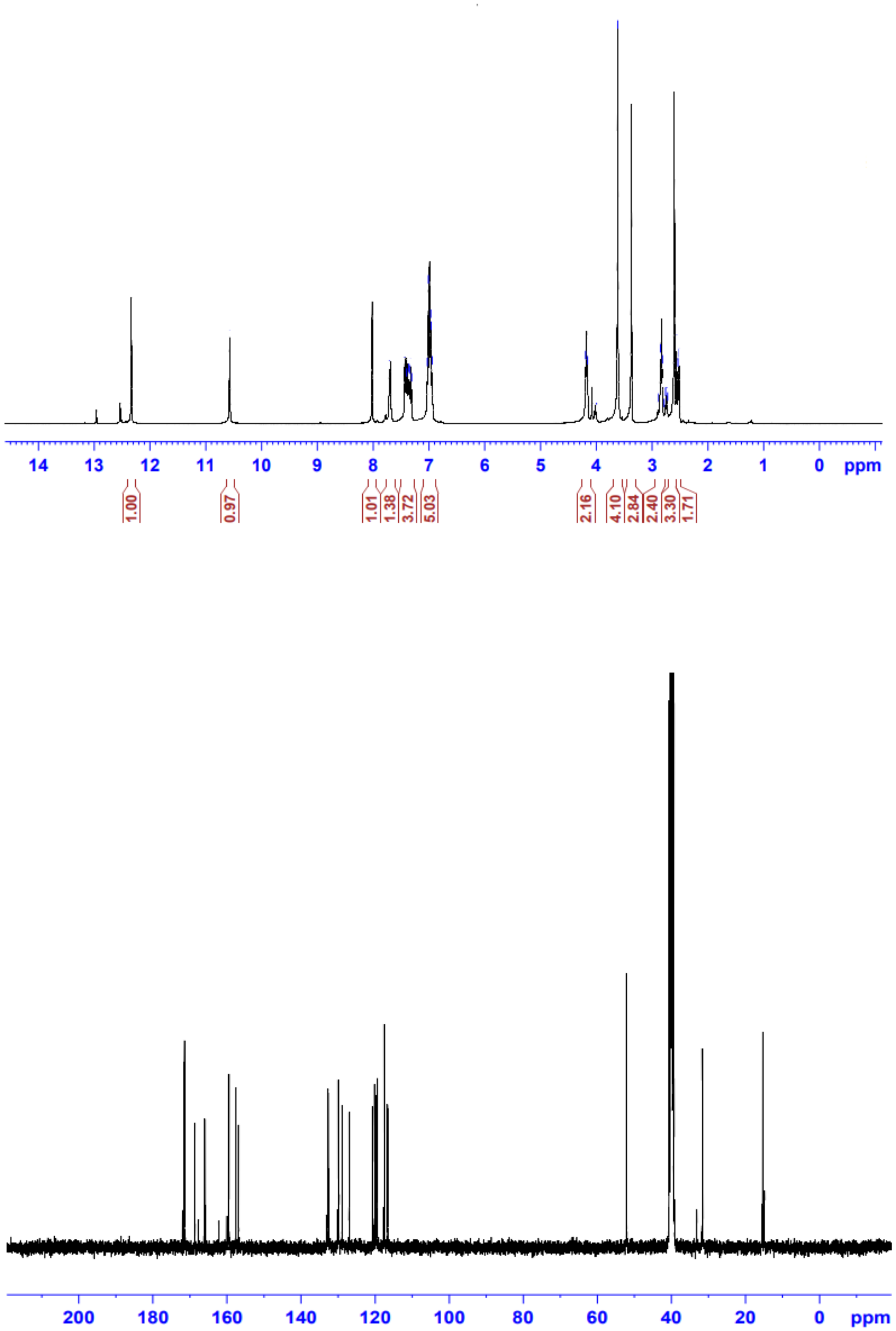

Figure S4a. ^{1}H **NMR and** ^{13}C **NMR spectra of compound 5a**

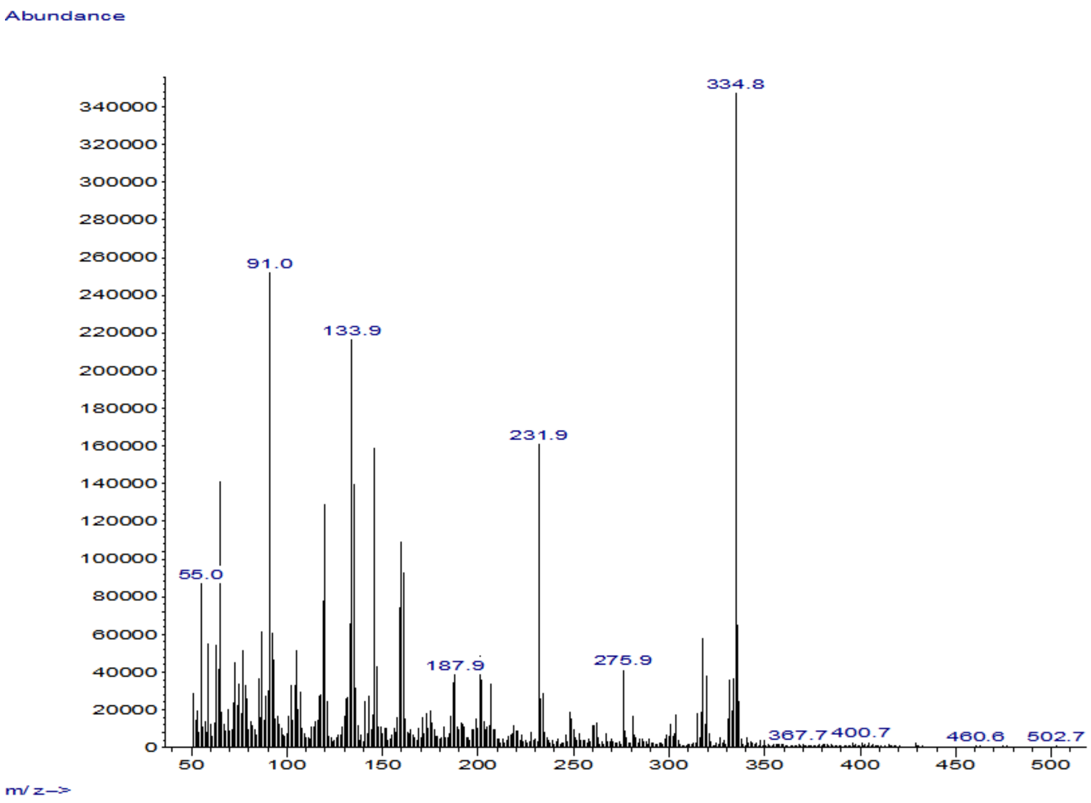

Figure S4b. Mass spectrum of compound 5a

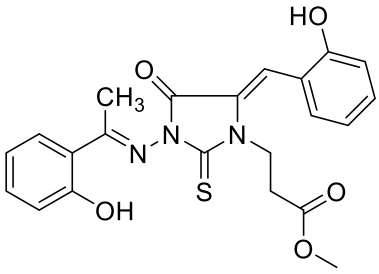

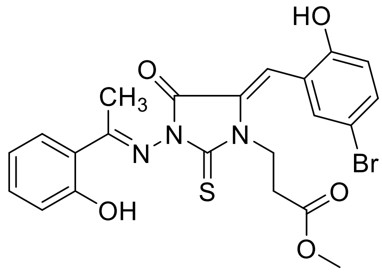

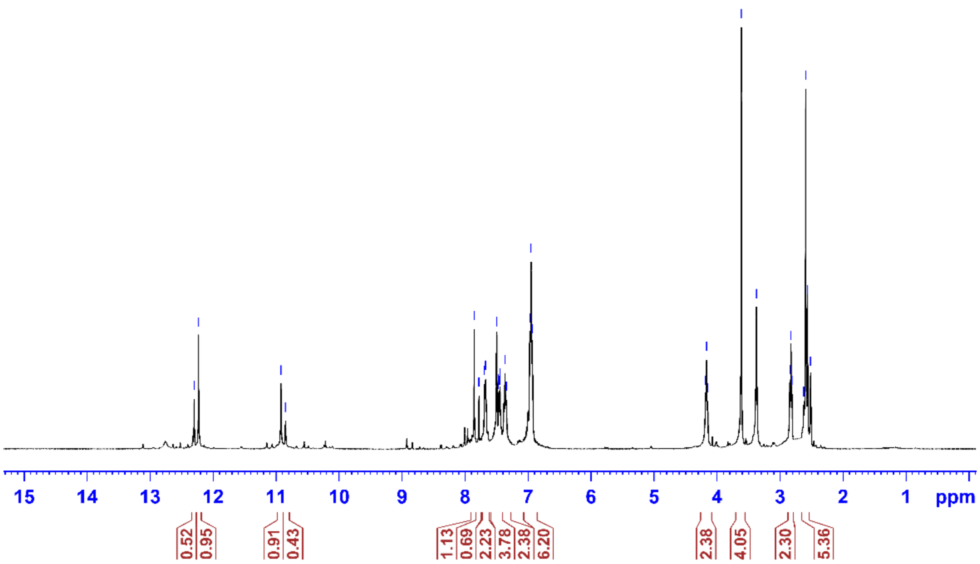

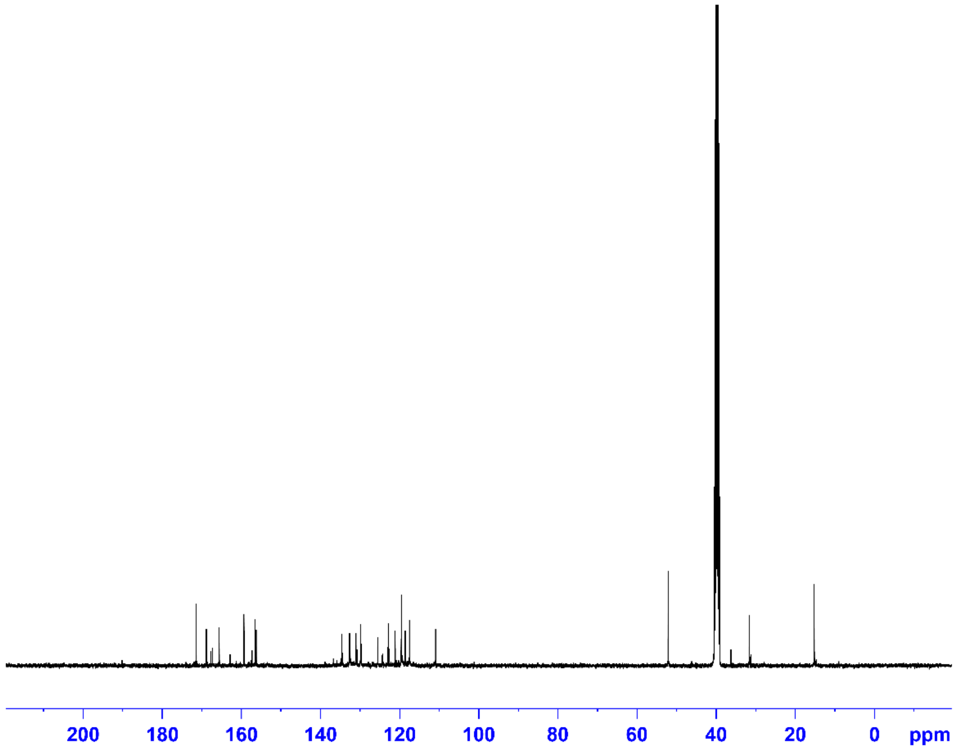

Figure S5. ^{1}**H NMR and** ^{13}**C NMR spectra of compound 5b**

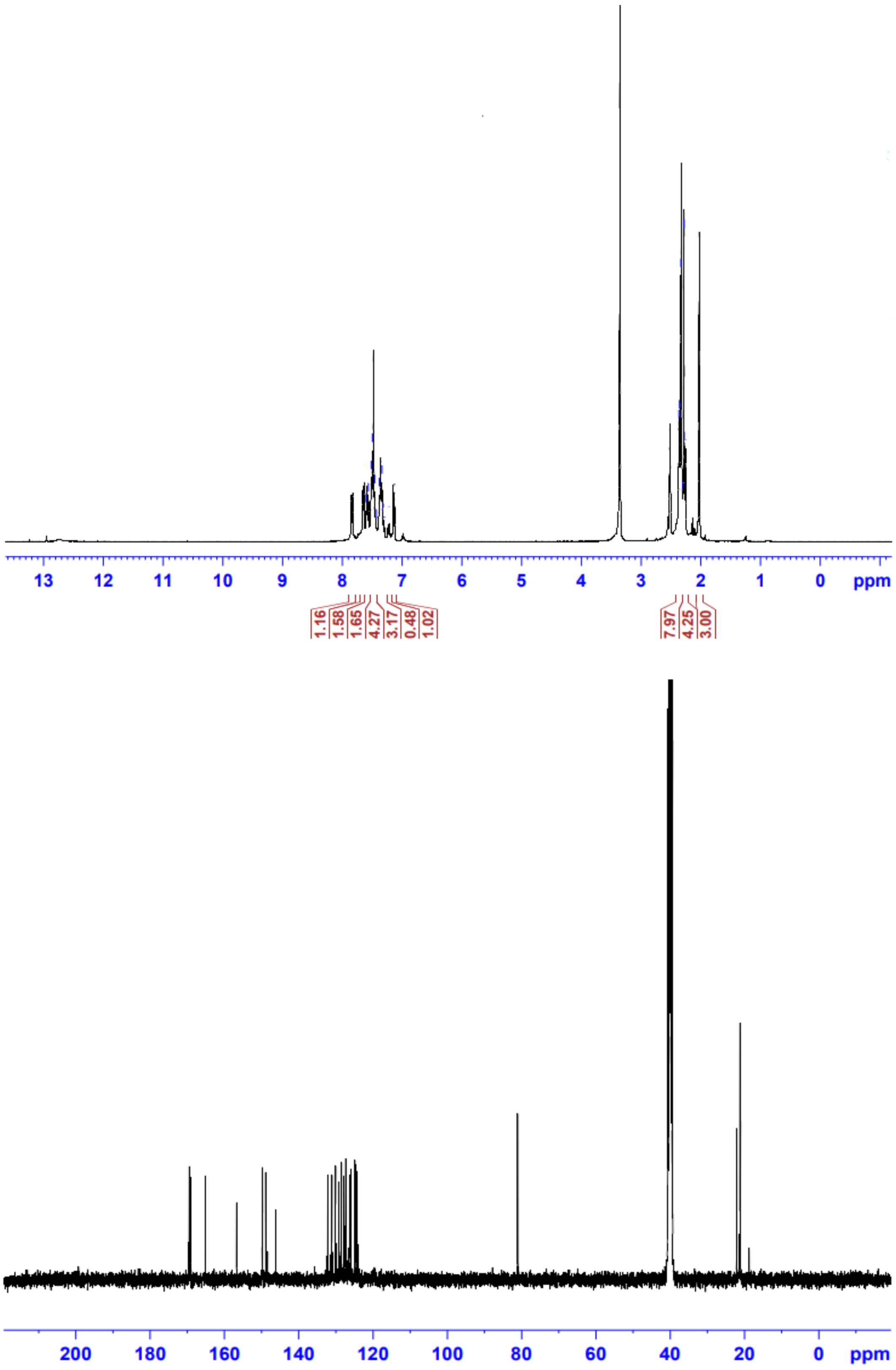

Figure S6a. ^{1}H NMR and ^{13}C NMR spectra of compound 6a

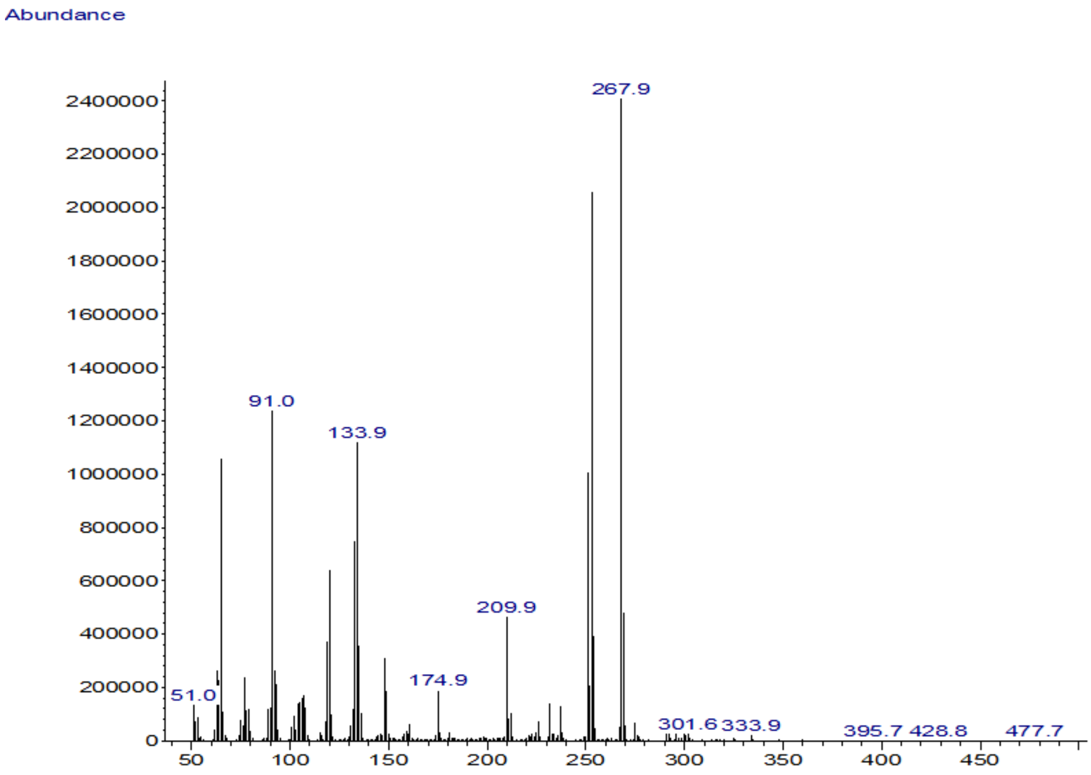

Figure S6a. Mass spectra of compound 6a

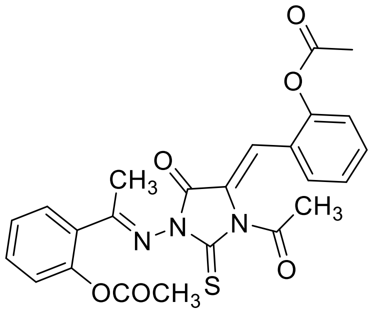

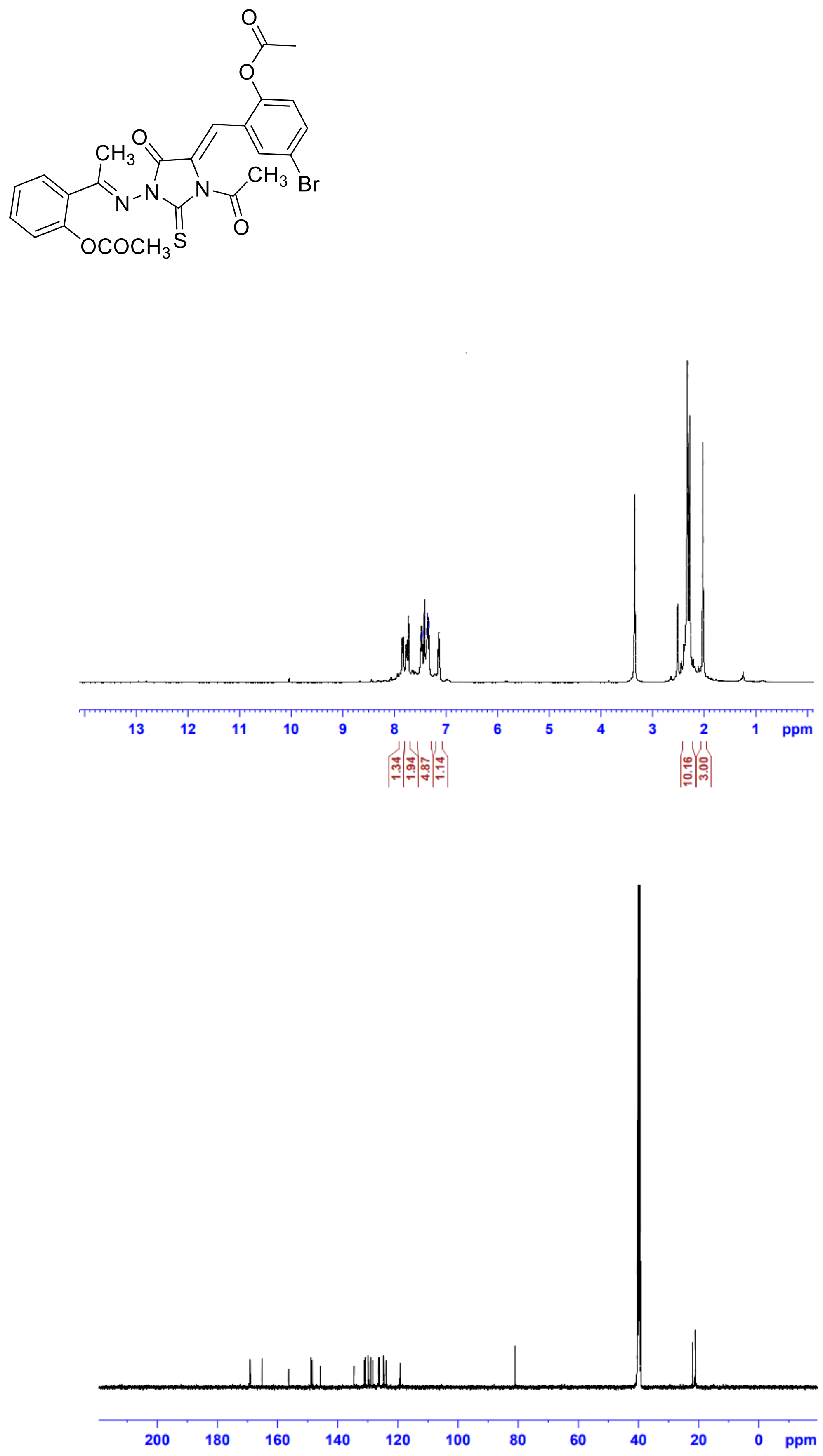

Figure S7. ^{1}**H NMR and** ^{13}**C NMR spectra of compound 6b**

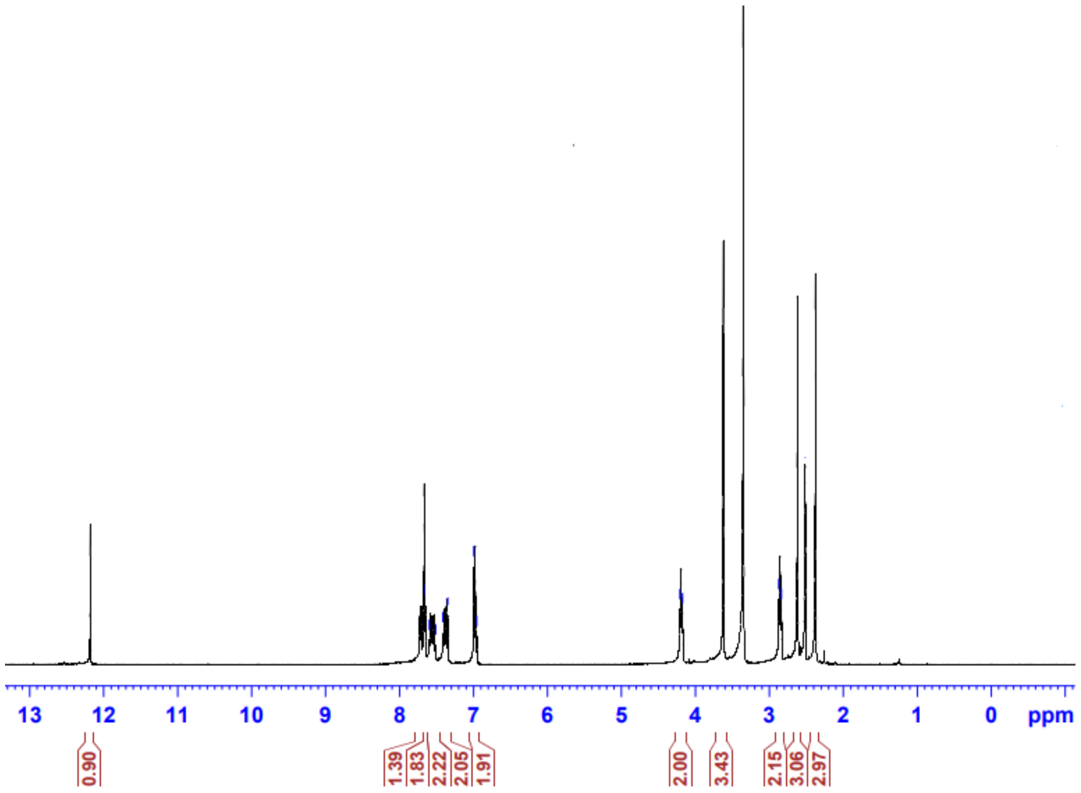

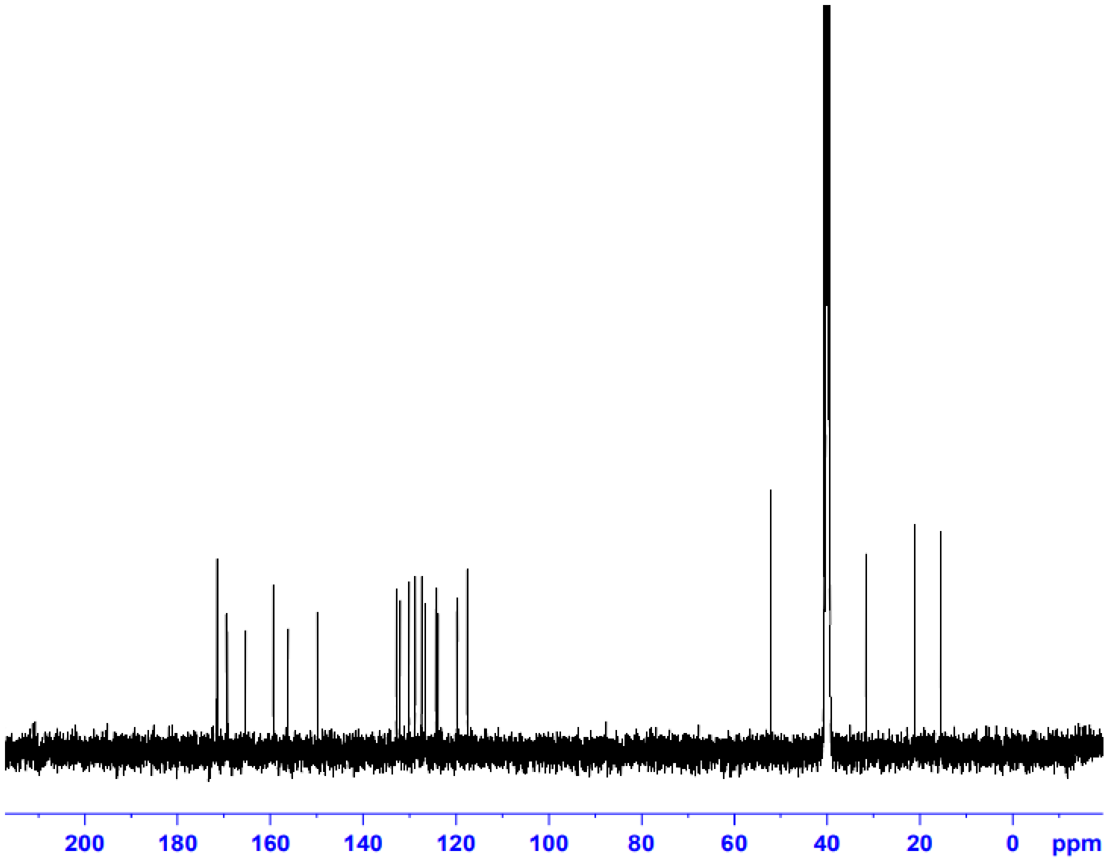

Figure S8a. ^{1}H **NMR and** ^{13}C **NMR spectra of compound 7a**

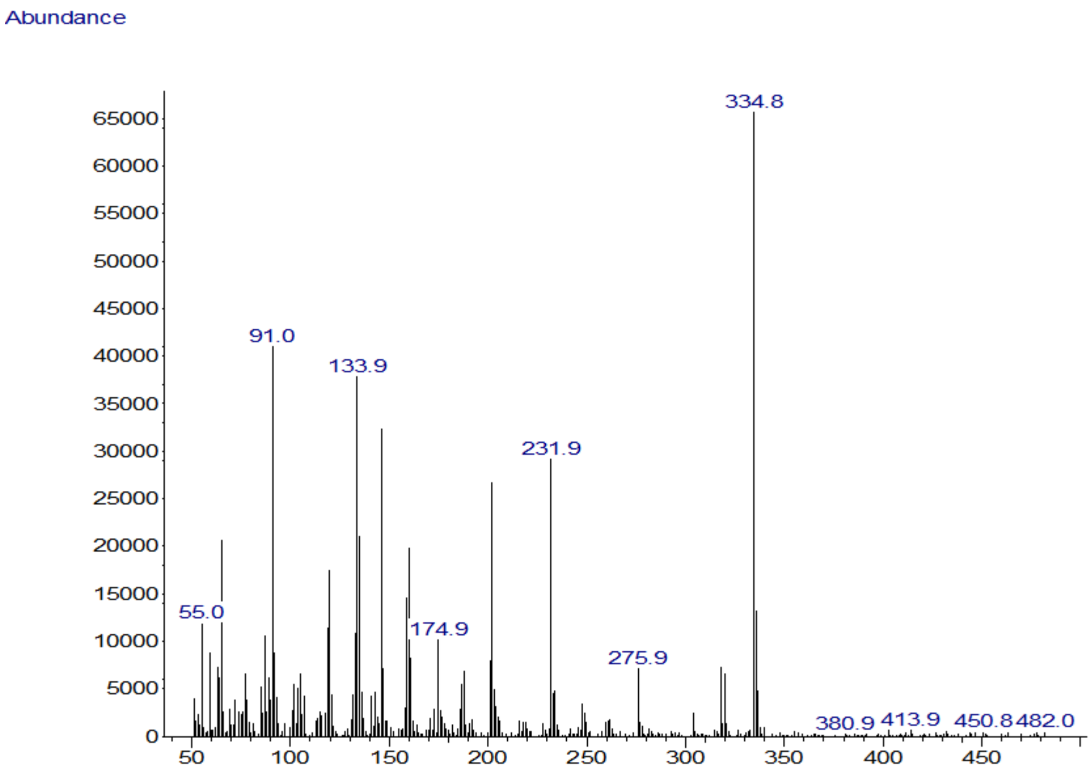

Figure S8b. Mass spectra of compound 7a

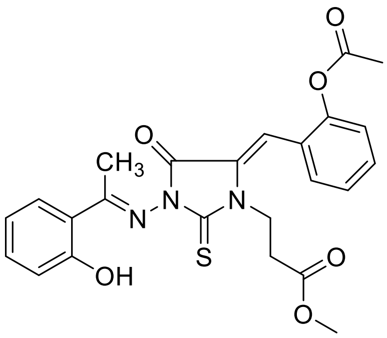

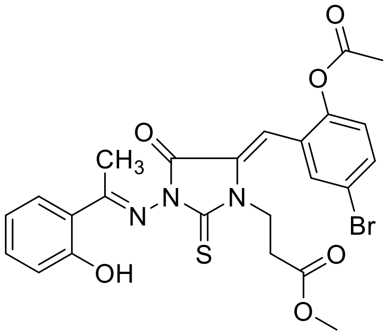

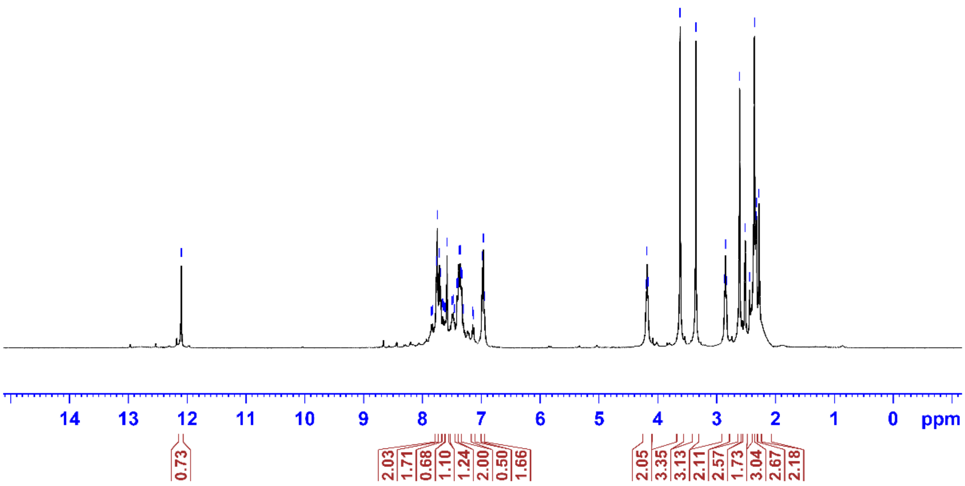

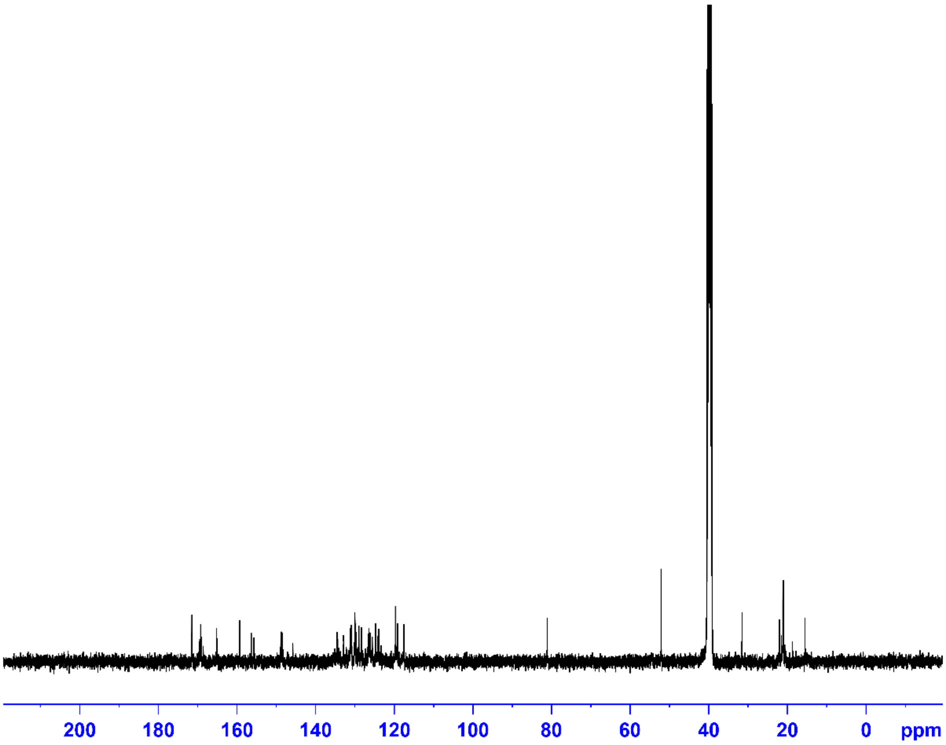

Figure S9. ^{1}H NMR and ^{13}C NMR spectra of compound 7b

Mass spectrometry investigation

The mass spectral fragmentation modes of the prepared 1,3,5-trisubstituted-imidazolidinones (**4a**, **5a**, **6a**, and **7a**) have been investigated. The mass spectra of compounds 4a-7a showed that the molecular ions of these compounds are unstable.

Compounds 4a and 6a:

Compounds 4a and 6a showed unstable molecular ions of these compounds in the mass spectra but underwent fragmentation with rearrangement to produce the stable ion peaks at m/z 257 and m/z 268, respectively (Scheme S1). The ion of m/z 353 of compounds 4a and 6a were also found to undergo fragmentation to produce the peak at m/z 134, 119, and m/z 91, respectively.

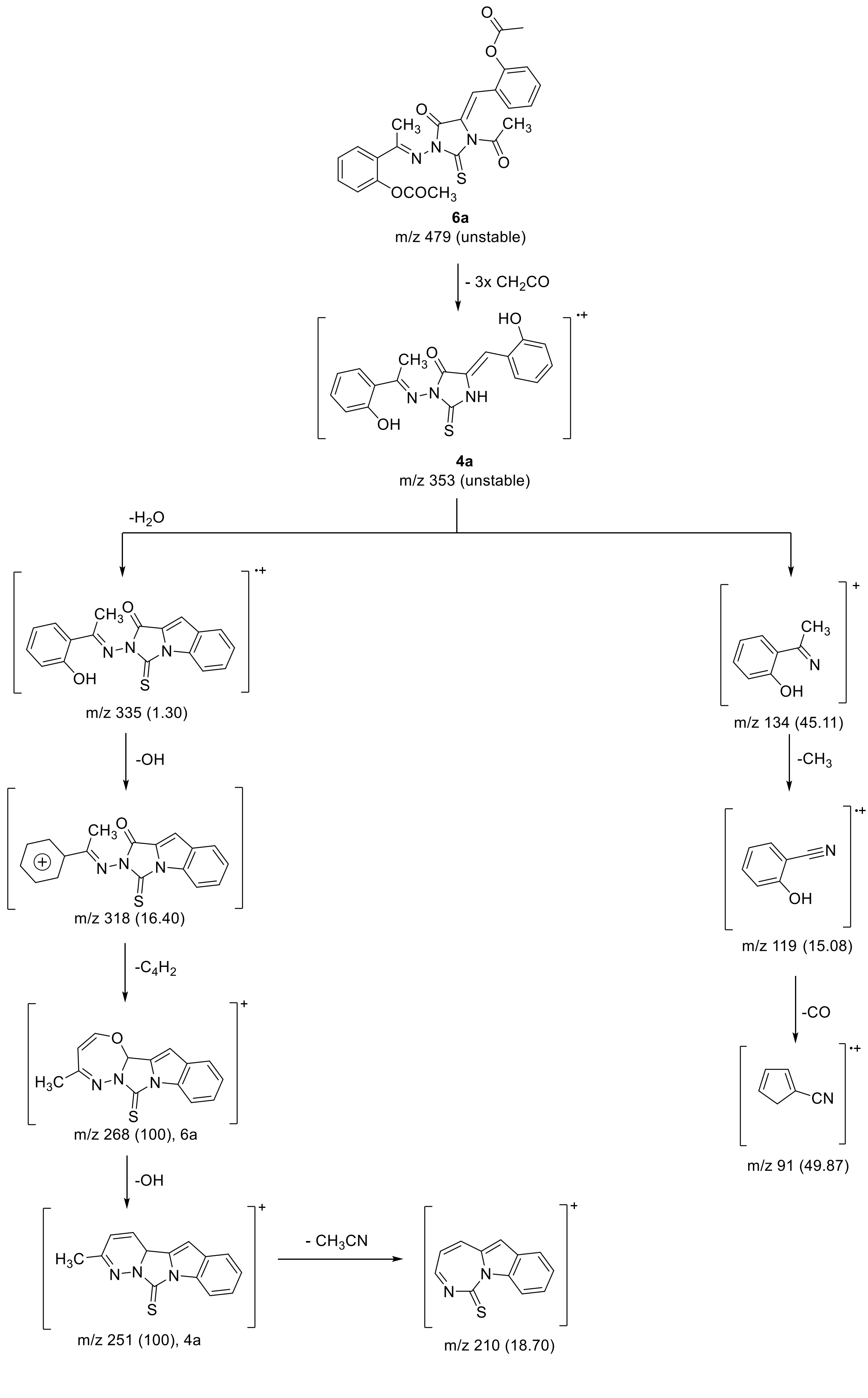

OCOCH3
6a
m/z 479 (unstable)
- 3x CH2CO
4a
m/z 353 (unstable)
-H2O
m/z 335 (1.30)
-OH
m/z 318 (16.40)
-C4H2
m/z 268 (100), 6a
-OH
m/z 251 (100), 4a
- CH3CN
m/z 210 (18.70)
m/z 134 (45.11)
-CH3
m/z 119 (15.08)
-CO
m/z 91 (49.87)

Scheme S1: The represented mass fragmentation pattern of compounds **4a** and **6a**

Compounds 5a and 7a:

The molecular ion peaks of compounds **5a** and **7a** were observed at m/z 439 and 481, respectively, unstable. The molecular ions of these compounds were fragmented with rearrangement to give stable ion peaks at m/z 335 (scheme S2). The stable ion peak at m/z 335 underwent fragmentation to produce the ion peak at m/z 134, 119, and 91, respectively. Further, the ion at m/z 353 underwent fragmentation to give ion peaks at m/z 260.

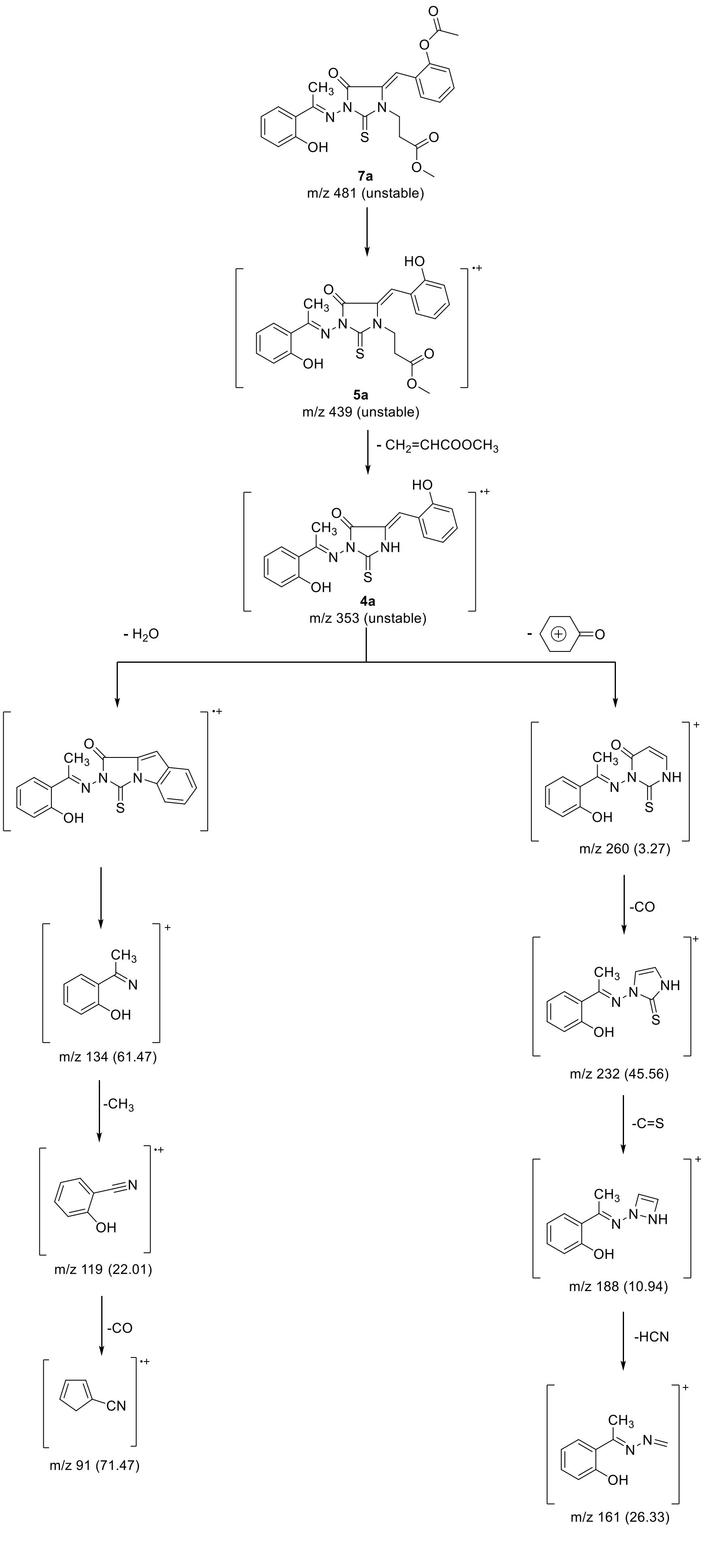

7a
m/z 481 (unstable)
5a
m/z 439 (unstable)
- CH2=CHCOOCH3
4a
m/z 353 (unstable)
- H2O
m/z 134 (61.47)
-CH3
m/z 119 (22.01)
-CO
m/z 91 (71.47)
m/z 260 (3.27)
-CO
m/z 232 (45.56)
-C=S
m/z 188 (10.94)
-HCN
m/z 161 (26.33)

Scheme S2: The represented mass fragmentation pattern of compounds **4a** and **6a**

Table S1: Inhibitory activities of 1,3,5-trisubstituted-2-thioxoimidazolidin-4-ones compounds toward α-glucosidase activity.

Compound 4a

log	%inh
3	83.1
2	62.6
1	44.9
0	24.5

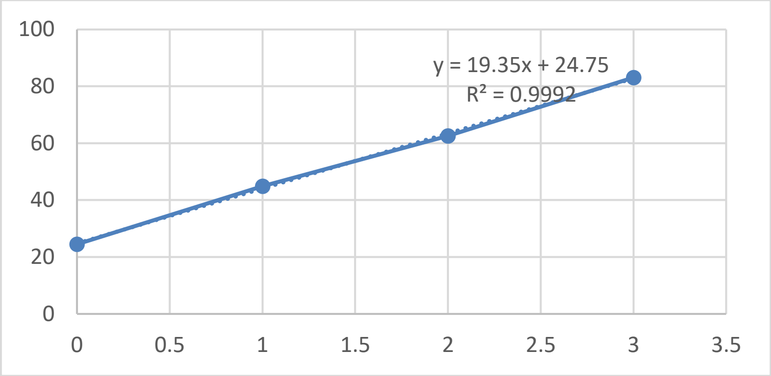

Compound4b

log	%inh
3	84.7
2	58.9
1	36.4
0	20.4

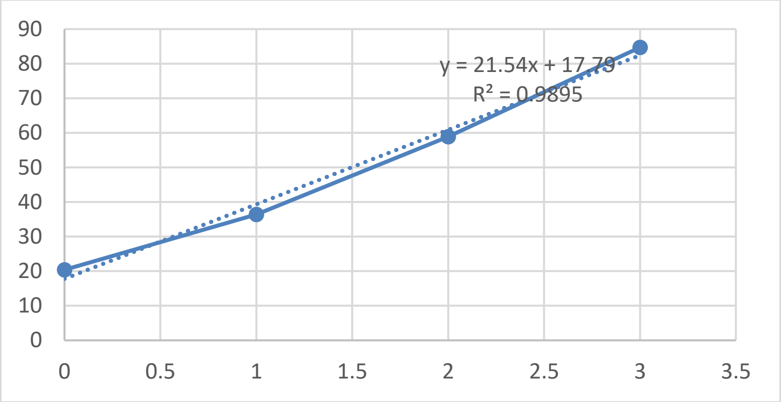

Compound 5a

log	%inh
3	86
2	75
1	58
0	35

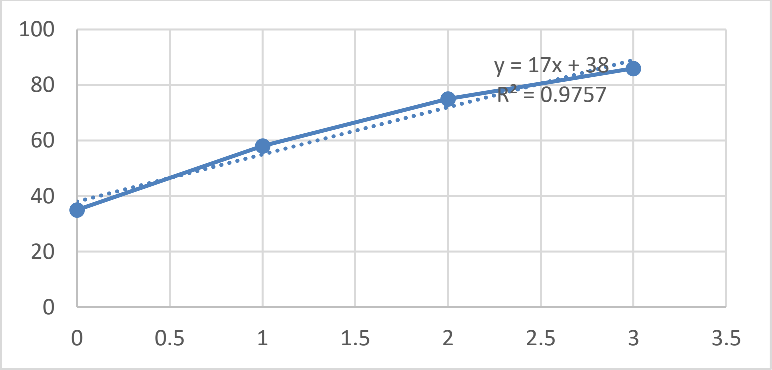

Compound 5b

log	%inh
3	87.4
2	71.4
1	50.3
0	27.6

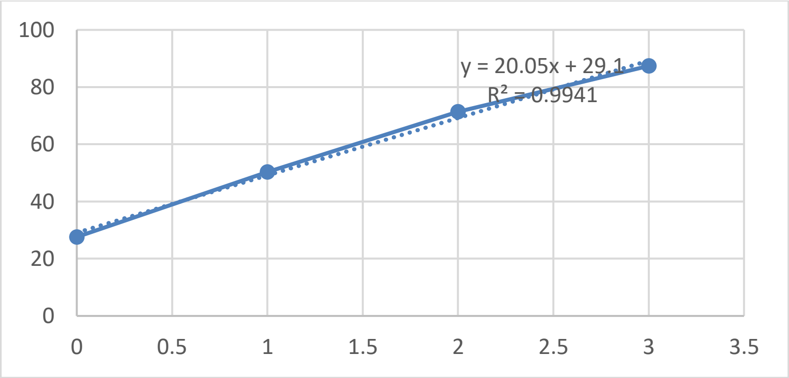

Compound 7a

log	%inh
3	82.6
2	66.1
1	48.3
0	21.8

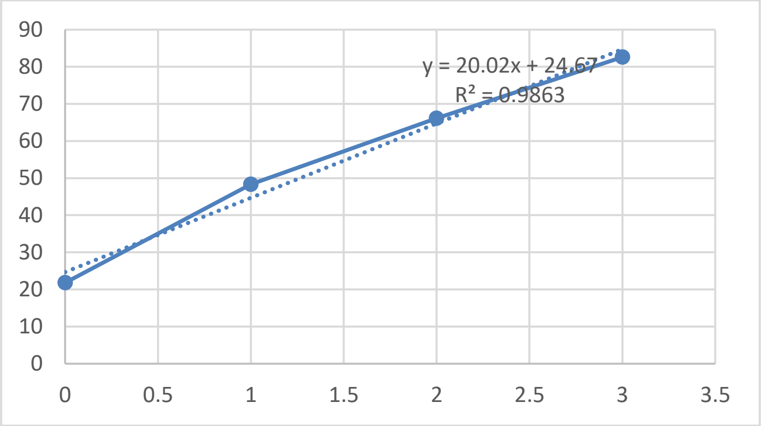

Compound 7b

log	%inh
3	86.5
2	70.4
1	47.8
0	28

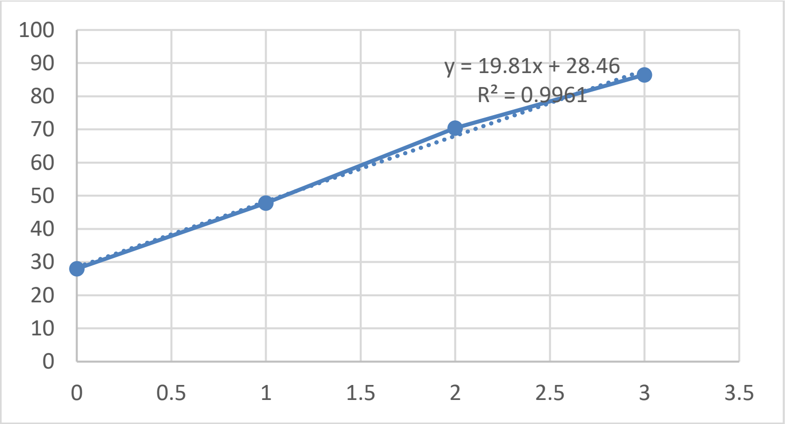

Acarbose

log	%inh
3	88.4
2	76.9
1	58.4
0	31.8

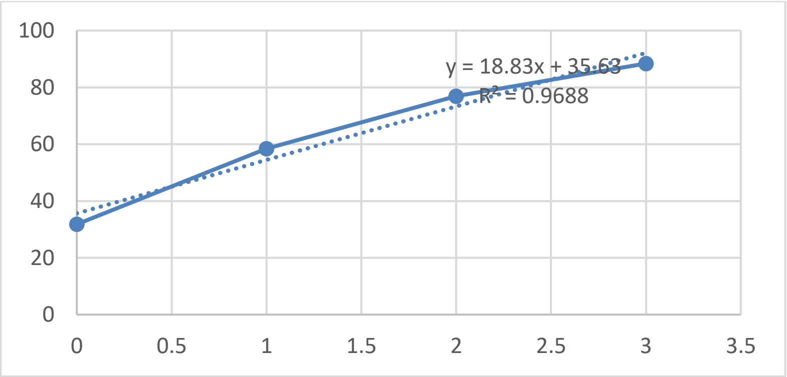

Table S2: Inhibitory activities of 1,3,5-trisubstituted-2-thioxoimidazolidin-4-ones derivatives toward α-amylase activity.

Compound 4a

log	%inh
2	81.9
1	61.4
0	44.1
-1	21.6

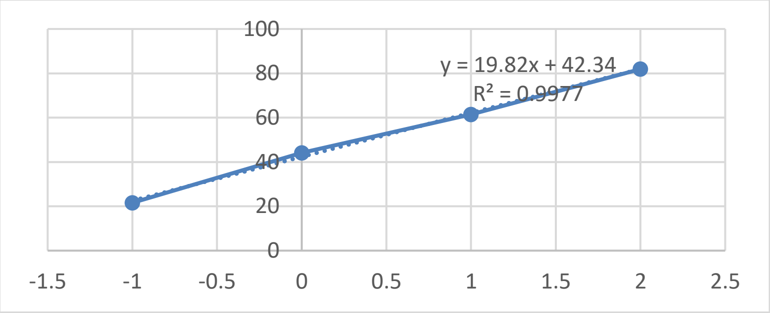

Compound 4b

log	%inh
2	81.4
1	58.7
0	30.1
-1	15.3

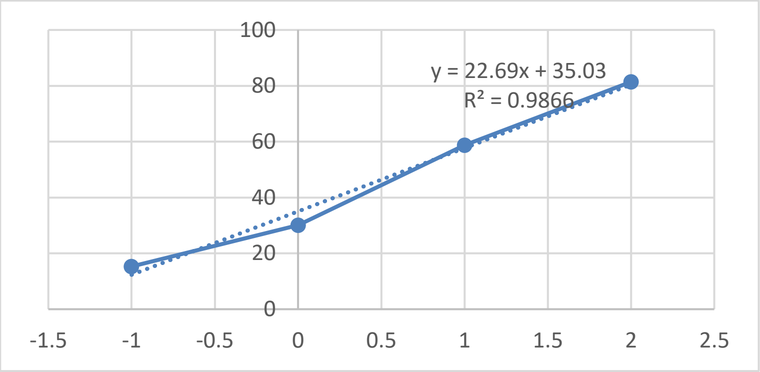

Compound 5a

log	%inh
2	88.8
1	77.9
0	63.4
-1	42.6

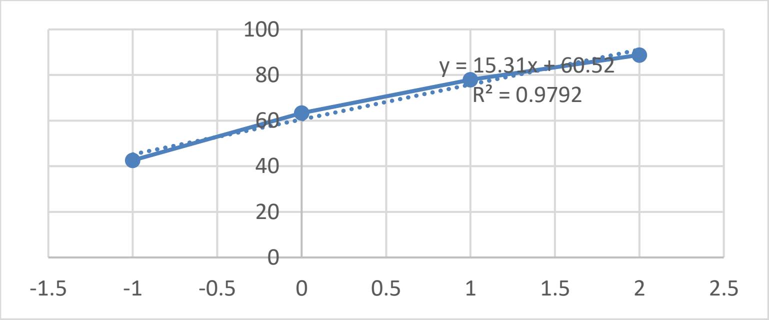

Compound 5b

log	%inh
2	84.7
1	67.5
0	53.1
-1	29

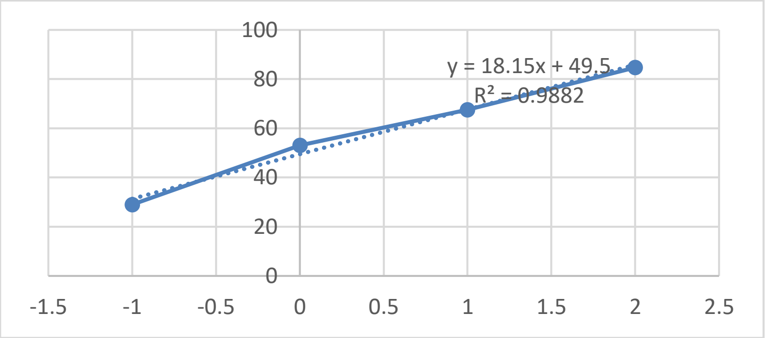

Compound 7a

log	%inh
2	84.8
1	76.6
0	54
-1	30.8

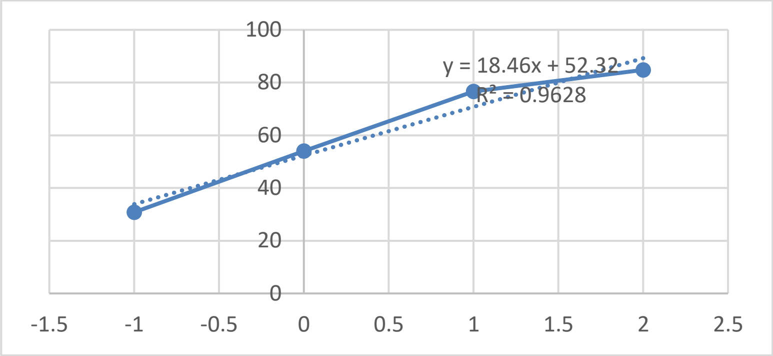

Compound 7b

log	%inh
2	87.6
1	65.2
0	43.3
-1	27.5

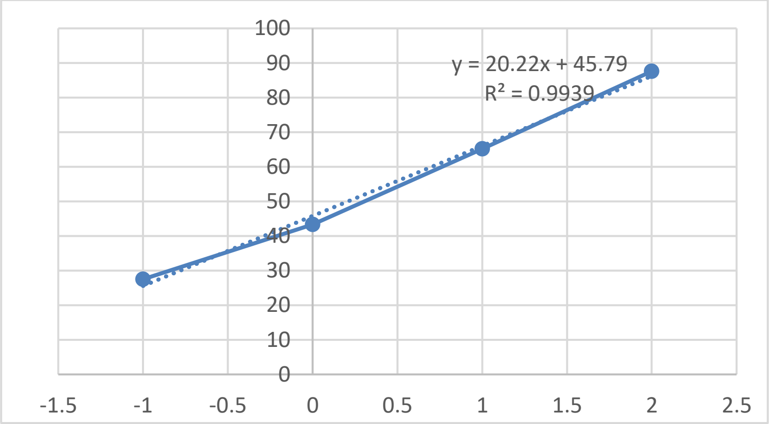

Acarbose

log	%inh
2	92.1
1	80.3
0	58.3
-1	37.2

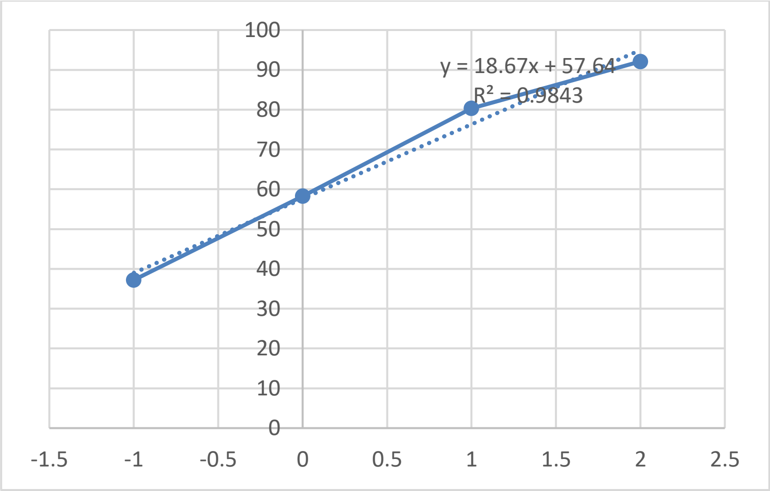

Table S3: In vitro cytotoxicity of compounds **(5a and 7a)** against WI-38 cells.

**Compounds**	**WI-38 Cytotoxicity IC50 ug/ml**	**SD ±**
	**WI-38**	
**5a**	88.538	3.92
**7a**	109.31	4.84
**Celecoxib**	93.054	4.12

Compound 5a

log conc.	% viability
3	34.56
2.398	44.07
1.799	52.91
1.204	59.79
0.602	69.02

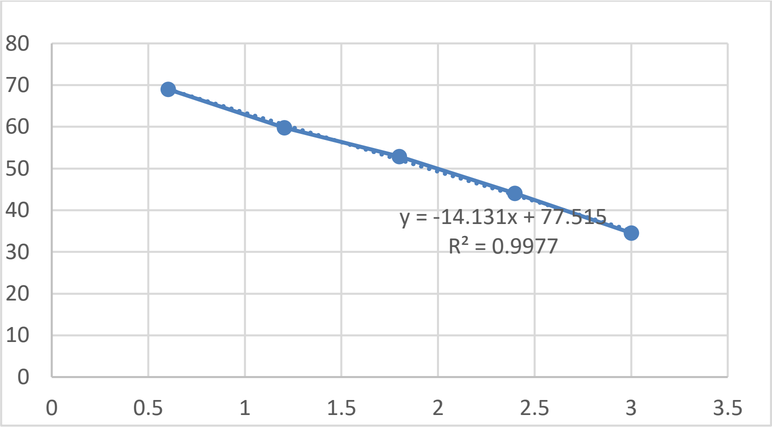

Compound 7a

log conc.	% viability
3	36.63
2.39794	44.07
1.79934	53.41
1.20412	60.63
0.60206	72.99

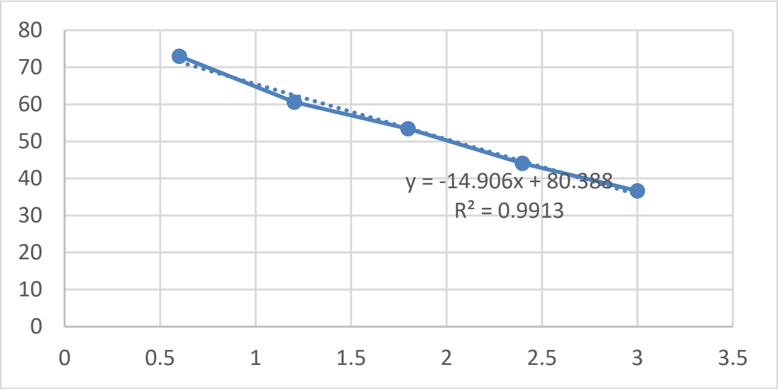

CXB/WI38

log conc.	% viability
3	35.4
2.398	42.99
1.799	54.24
1.204	61.66
0.602	67.26

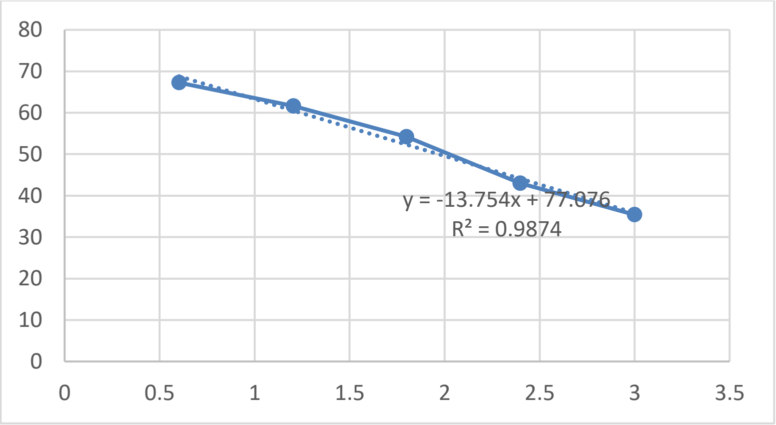

Table S4: Effect of Compounds (**5a** and **7a**) on scavenging DPPH free radical.

Compound 5a

conc	%RSA
200	90.9673
100	84.0278
50	65.3256
25	49.2589
12.5	34.2
6.25	25.9268
3.12	12.8385

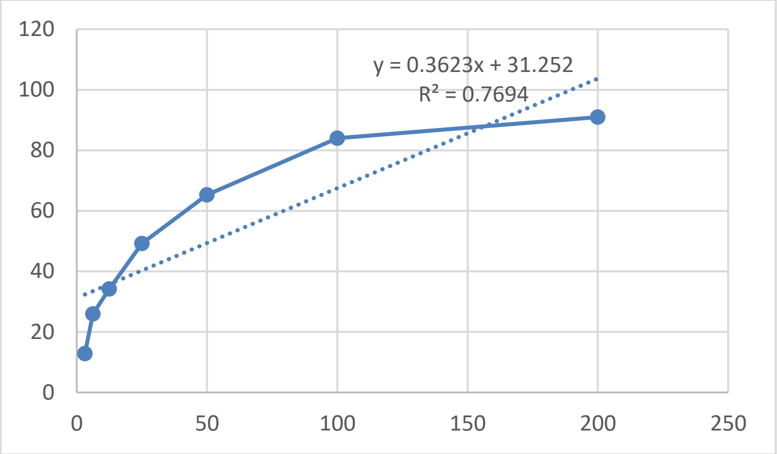

Compound 7a

conc	%RSA
200	80.732
100	81.6087
50	62.2992
25	45.4635
12.5	30.2839
6.25	18.9636
3.12	9.7382

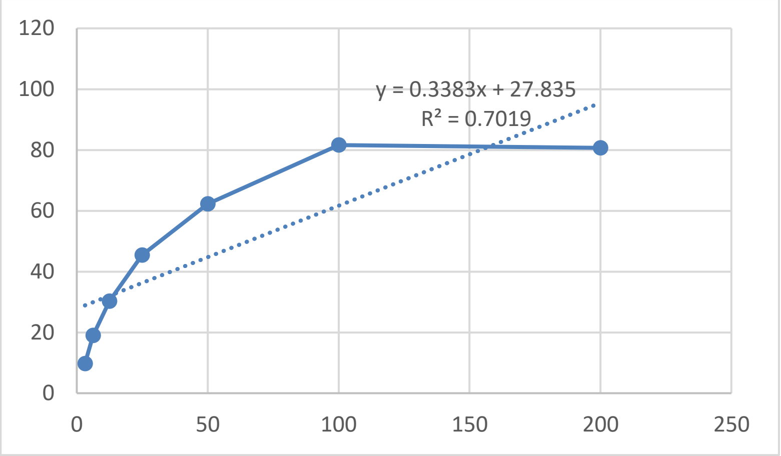

Torolox

conc	%RSA
200	89.0026
100	82.6087
50	63.2992
25	47.4425
12.5	32.4808
6.25	20.2046
3.12	11.3811

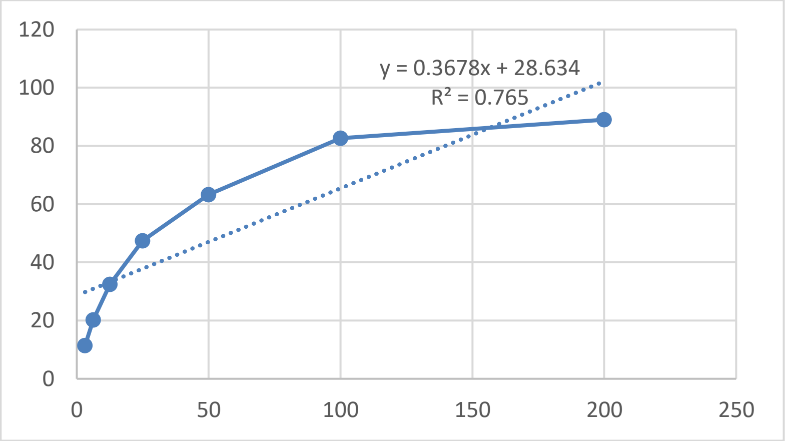

Compounds	Scavenging free radical DPPH IC50 ug/ml	SD ±
**5a**	51.75	**5.2**
**7a**	65.5	**5.6**
**Torolox**	58.09	**3.1**

Table S5: Effect of Compounds (**5a** and **7a**) on ROS generation.

**Compounds**	**ROS Pg/ml**
**5a**	**132.4±2.16**
**7a**	**191.5±3.62**
**Celecoxib**	**171.6±1.89**
**Control**	**90.74±5.54**
